# Integrative genomic analysis implicates *ERCC6* and its interaction with *ERCC8* in susceptibility to breast cancer

**DOI:** 10.1038/s41598-020-77037-7

**Published:** 2020-12-04

**Authors:** Roxana Moslehi, Hui-Shien Tsao, Nur Zeinomar, Cristy Stagnar, Sean Fitzpatrick, Amiran Dzutsev

**Affiliations:** 1 School of Public Health, Cancer Research Center, University at Albany, State University of New York (SUNY), Albany, NY 12144 USA; 2 New York State Office of Children and Family Services, New York, USA; 3 Mailman School of Public Health, Columbia University, New York, USA; 4 Drukier Institute for Children’s Health, Weill Cornell Medicine, New York, USA; 5 Cancer Vaccine Branch, National Cancer Institute, National Institutes of Health, Bethesda, MD USA

**Keywords:** Cancer, Genetics, Systems biology

## Abstract

Up to 30% of all breast cancer cases may be inherited and up to 85% of those may be due to segregation of susceptibility genes with low and moderate risk [odds ratios (OR) ≤ 3] for (mostly peri- and post-menopausal) breast cancer. The majority of low/moderate-risk genes, particularly those with minor allele frequencies (MAF) of < 30%, have not been identified and/or validated due to limitations of conventional association testing approaches, which include the agnostic nature of Genome Wide Association Studies (GWAS). To overcome these limitations, we used a hypothesis-driven integrative genomics approach to test the association of breast cancer with candidate genes by analyzing multi-omics data. Our candidate-gene association analyses of GWAS datasets suggested an increased risk of breast cancer with *ERCC6* (main effect: 1.29 ≤ OR ≤ 2.91, 0.005 ≤ p ≤ 0.04, 11.8 ≤ MAF ≤ 40.9%), and implicated its interaction with *ERCC8* (joint effect: 3.03 ≤ OR ≤ 5.31, 0.01 ≤ p_interaction_ ≤ 0.03). We found significant upregulation of *ERCC6* (p = 7.95 × 10^–6^) and *ERCC8* (p = 4.67 × 10^–6^) in breast cancer and similar frequencies of *ERCC6* (1.8%) and *ERCC8* (0.3%) mutations in breast tumors to known breast cancer susceptibility genes such as *BLM* (1.9%) and *LSP1* (0.3%). Our integrative genomics approach suggests that *ERCC6* may be a previously unreported low- to moderate-risk breast cancer susceptibility gene, which may also interact with *ERCC8*.

## Introduction

Breast cancer is the most commonly-reported cancer and the second leading cause of cancer death among women in the United States (US). Approximately, 1 in 8 women in the US will develop breast cancer in their lifetime and 1 in 35 will die from the disease^1^. There will be an estimated 276,480 new cases of invasive breast cancer and 42,170 deaths due to breast cancer among US women in 2020^1^. Breast cancer is a complex disorder with genetic and environmental factors playing synergistic roles in its etiology.

**Figure 1:**
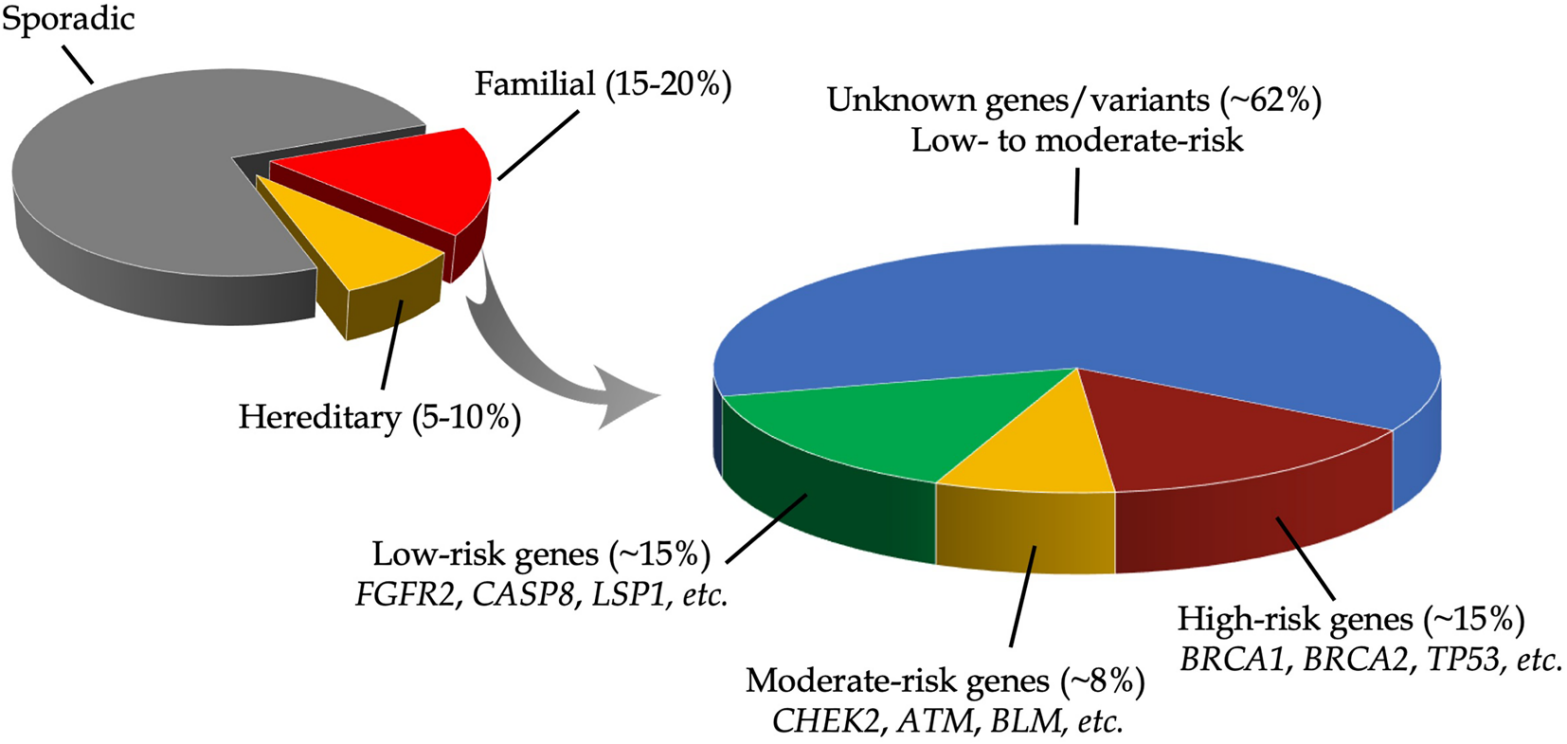
Breast cancer susceptibility.

Up to 30% of all breast cancer cases in the general population may be due to inherited susceptibility factors^2^. About 15% of inherited breast cancer cases may be due to segregation of rare germline mutations [minor allele frequency (MAF) ≤ 1%] in high-penetrance [i.e., relative risk (RR) > 10] susceptibility genes such as *BRCA1*, *BRCA2*, *TP53*, and *PTEN*^2,3^. Medium-penetrance (3 ≤ RR ≤ 10) genes such as *CHEK2*, *ATM, PALB2* (suggested by some as being high-penetrance^4,5^), *BRIP1* (disputed as a breast cancer susceptibility gene by some^6^) have also been identified and account for about 8% of inherited breast cancers cases^2^. Nearly all high- and medium-penetrance genes were identified through family-based genetic epidemiologic investigations and gene resequencing^7^. Early (pre-menopausal) onset of the disease is a well-established hallmark of inherited breast cancers that are due to segregation of mutations in high- and medium-penetrance genes. Low-penetrance [RR or odds ratio (OR) < 3] genes, such as *FGFR2*, *CASP8*, *MAP3K1* and *LSP1*, with MAF of about 30% in the general population and accounting for about 15% of inherited breast cancer cases have also been identified through genome-wide association studies (GWAS)^7^. Susceptibility genes accounting for the remaining of about 62% of inherited breast cancers (mostly believed to be low- to medium-penetrance genes leading to peri- or post-menopausal breast cancer) are yet to be identified and/or validated (Fig. 1).

Inherent limitations of GWAS, namely their agnostic nature necessitating stringent p-value thresholds, multiple-testing corrections, and exceedingly large sample size requirements for detecting small effects and gene–gene interactions, may be responsible for the apparent inability to identify and replicate the remainder of low- to medium-penetrance breast cancer susceptibility genes, particularly those with MAF < 30%. Integrative approaches enable extraction of deeper biological insights than what can be achieved through single-dimensional or conventional analyses. Inspired by our hypothesis-driven integrative genetic epidemiologic investigations of DNA repair disorders, which enabled us to decipher the biologic mechanisms that underlie the association between mutations in a subset of DNA repair genes and certain clinical outcomes associated with these disorders^8,9^, we designed a similar hypothesis-driven integrative approach to identify susceptibility genes and gene variants that influence the risk of common (i.e., peri- and post-menopausal) breast cancer. We report on the analysis of raw multiomics data from several relevant GWAS, transcriptome, and somatic mutation datasets to test the association of breast cancer with two candidate genes, *ERCC6* and *ERCC8*.

## Results

### Candidate gene analysis of genome-wide association studies (GWAS) datasets

#### Cancer Genetic Markers of Susceptibility (CGEMS)

The demographic characteristics of subjects in the CGEMS^10,11^ dataset analyzed in our study (1089 cases, 1093 controls) are described in the Methods section and were published in our previous report^12^. All *ERCC6* SNPs analyzed in CGEMS were in Hardy–Weinberg Equilibrium (HWE) and in strong Linkage Disequilibrium (LD) among the controls (Supplementary Fig. 1A). Both *ERCC8* SNPs were also in HWE and their LD pattern among the controls is shown in Supplementary Fig. 1B.

Three *ERCC6* SNPs, rs3750751 (NC_000010.11:g.49457882C > T), rs3750749 (NC_000010.11:g.49476182A > G), and rs4253082 (NC_000010.11:g.49509540C > T), were associated with an increased risk for breast cancer in CGEMS (Table 1). Statistically significant increased risks were found for homozygotes for the variant allele at rs3750751 (OR 2.91, 95% CI 1.05–8.06, p = 0.04), heterozygotes at rs3750749 (OR 1.40, 95% CI 1.07–1.82, p = 0.01), and heterozygotes at rs4253082 (OR 1.32, 95% CI 1.09–1.61, p = 0.005). The trend (i.e., increasing number of variant alleles) was also significant (OR 1.36, 95% CI 1.05–1.75, p = 0.02) for rs3750749 (Table 1).

**Table 1:** *ERCC6* and *ERCC8* single nucleotide polymorphisms (SNPs) significantly associated with breast cancer in at least one breast cancer genome-wide association study (GWAS) dataset used as secondary data in our study.

SNP		Alleles	Odds ratio (OR), 95% confidence interval (CI), p-value							
			CGEMS^a^		NSABP^b^		WHI^c^		BPC3^d^	
			Case (N = 1089)	Control (N = 1093)	Case (N = 430)	Control (N = 822)	Case (N = 465)	Control (N = 1394)	Case (N = 977)	Control (N = 1026)
rs3750751	0	GG	1.00		1.00		1.00		1.00	
	1	GA	0.99 (0.79–1.25), 0.96		0.63 (0.45–0.88), 0.01		NA		1.35 (1.01–1.80), 0.04	
	2	AA	2.91 (1.05–8.06), 0.04		2.53 (0.59–10.97), 0.21		NA		2.90 (0.52–16.07), 0.22	
Trend			1.10 (0.89–1.35), 0.37		0.74 (0.54–1.00), 0.05		NA		1.38 (1.05–1.81), 0.02	
rs2229760	0	GG	1.00		1.00		1.00		1.00	
	1	GA	1.08 (0.89–1.31), 0.43		1.40 (1.08–1.83), 0.01		NA		0.88 (0.70–1.09), 0.24	
	2	AA	1.03 (0.80–1.33), 0.80		1.28 (0.90–1.81), 0.16		NA		1.15 (0.84–1.56), 0.39	
Trend			1.03 (0.91–1.16), 0.68		1.17 (0.99–1.38), 0.07		NA		1.02 (0.88–1.19), 0.76	
rs3750749	0	TT	1.00		1.00		1.00		1.00	
	1	TC	1.40 (1.07–1.82), 0.01		1.00 (0.69–1.46), 1.00		NA		0.86 (0.64–1.15), 0.31	
	2	CC	1.06 (0.21–5.28), 0.94		0.40 (0.05–3.43), 0.40		NA		0.20 (0.02–1.73), 0.14	
Trend			1.36 (1.05–1.75), 0.02		0.94 (0.66–1.34), 0.74		NA		0.81 (0.61–1.07), 0.14	
rs4253082	0	GG	1.00		1.00		1.00		1.00	
	1	GA	1.32 (1.09–1.61), 0.005		1.04 (0.80–1.34), 0.79		1.27 (0.99–1.64), 0.06		1.24 (0.64–2.38), 0.52	
	2	AA	0.79 (0.44–1.41), 0.43		0.81 (0.34–1.93), 0.64		0.98 (0.45–2.11), 0.96		1.27 (0.67–2.40), 0.46	
Trend			1.17 (0.99–1.39), 0.06		0.91 (0.71–1.16), 0.44		NA		1.05 (0.87–1.28), 0.59	
rs2228528	0	GG	1.00		1.00		1.00		1.00	
	1	GA	NA		1.04 (0.80–1.35), 0.77		1.29 (1.01–1.66), 0.04		0.99 (0.79–1.24), 0.91	
	2	AA	NA		0.82 (0.35–1.93), 0.64		1.02 (0.47–2.21), 0.95		0.82 (0.43–1.56), 0.55	
Trend			NA		1.00 (0.80–1.26), 0.98		NA		0.96 (0.79–1.16), 0.69	
rs1012553	0	TT	1.00		1.00		1		1.00	
	1	TA	NA		NA		1.35 (1.07–1.71), 0.01		NA	
	2	AA	NA		NA		1.13 (0.69–1.84), 0.63		NA	
Trend			NA		NA		1.20 (1.00–1.44), 0.05		NA	

*NA* Not analyzed due to missing SNPs or subjects (i.e., cells containing 0 subjects).

^a^Cancer Genetic Markers of Susceptibility^10,11^: Our analysis of raw data involved Caucasian women ≥ 55 years of age using unconditional logistic regression adjusting for family history of breast cancer.

^b^National Surgical Adjuvant Breast and Bowel Project (NSABP) Prevention Trials (P-1^14^ and P-2^13^): Our analysis of raw data involved Caucasian women ≥ 50 years of age using conditional logistic regression maintaining matching criteria set by the original study investigators (i.e., age at trial entry, time in the study, history of lobular carcinoma in situ, and 5-year predicted breast cancer risk based on the Gail model).

^c^Women's Health Initiative (WHI)^15,16^ Hormone Therapy Trials data was used to create a nested case–control dataset of women diagnosed with invasive breast cancer ≥ 50 years of age (N = 465) and healthy controls (N = 1394) frequency-matched to the cases based on age in 3:1 control to case ratio: Our analysis of raw data involved Caucasian women ≥ 50 years of age using unconditional logistic regression adjusting for family history of breast cancer, parity, oral contraceptive use, breast feeding and body mass index.

^d^Breast and Prostate Cancer Cohort Consortium (BPC3)^17,18^: Our analysis of raw data involved Caucasian women ≥ 50 years of age using unconditional logistic regression adjusting for family history of breast cancer.

Haplotype analysis revealed borderline significant association of breast cancer with *ERCC6* Hap 5 (OR 1.30, 95% CI 1.00–1.69, p = 0.048) (Supplementary Table 2). Diplotype analysis revealed statistically-significant association of breast cancer with three diplotypes in *ERCC6;* these included 2/5 (OR 2.65, 95% CI 1.14–6.17, p = 0.024), 1/6 (OR 3.89, 95% CI 1.42–10.66, p = 0.008), and 4/4 (OR 2.92, 95% CI 1.04–8.22, p = 0.042) (Supplementary Table 2).

None of the *ERCC8* SNPs, haplotypes or diplotypes were associated with a statistically significant increased risk of breast cancer in CGEMS. Joint effect analysis revealed increased risk of breast cancer with the *ERCC8* 1/1 and *ERCC6* 2/5 diplotype combination (OR 3.03, 95% CI 1.16–7.91, p = 0.024) and the *ERCC8* 1/1 and *ERCC6* 1/6 diplotype combination (OR 3.27, 95% CI 1.17, 9.18, p = 0.024) compared with the reference category (Table 2A). Gene–gene interaction analysis revealed statistically-significant interaction between *ERCC6* and *ERCC8* at the diplotype level (p_interaction_ = 0.010) (Table 2A).

**Table 2:** Joint effect analysis of *ERCC6* and *ERCC8* diplotypes.

*ERCC8*	A. Cancer Genetic Markers of Susceptibility (CGEMS)																	
	*ERCC6*																	
	Hap0/Hap0						Hap2/Hap5						Hap1/Hap6					
	Control		Case		Adjusted OR (95% CI)	p-Value	Control		Case		Adjusted OR (95% CI)	p-Value	Control		Case		Adjusted OR (95% CI)	p-Value
	n	%	n	%			n	%	n	%			n	%	n	%		
Hap1/Hap1	162	78.3	163	69.10	1		**6**	**2.9**	**17**	**7.2**	**3.03 (1.16–7.91)**	**0.024**	**5**	**2.4**	**16**	**6.8**	**3.27 (1.17–9.18)**	**0.024**
Hap1/Hap2	18	8.7	9	3.80	0.50 (0.22, 1.14)	0.100	1	0.5	1	0.4	1.11 (0.07,17.89)	0.943					NA	NA
Hap1/Hap3	9	4.3	15	6.40	1.72 (0.73, 4.05)	0.217	1	0.5	2	0.8	1.92 (0.17,21.59)	0.599					NA	NA

*ERCC8*	B. Women’s Health Initiative (WHI) Hormone Therapy Trials																	
	*ERCC6*																	
	Hap0/Hap0						Hap2/Hap4											
	Control		Case		Adjusted OR (95% CI)	p-Value	Control		Case		Adjusted OR (95% CI)	p-value						
	n	%	n	%			n	%	n	%								
Hap0/Hap0	81	41.10	20	29.00	1		**5**	**2.50**	**5**	**7.20**	**5.31 (1.22–23.09)**	**0.026**						
Hap0/Hap1	34	17.30	18	26.10	2.13 (0.95**–**4.77)	0.066	2	1.00	1	1.40	2.23 (0.18**–**27.51)	0.533						
Hap0/Hap2	25	12.70	10	14.50	2.06 (0.80**–**5.30)	0.136	3	1.50	1	1.40	1.47 (0.14**–**15.84)	0.749						
Hap0/Hap3	23	11.70	4	5.80	0.97 (0.28**–**3.33)	0.965	3	1.50	3	4.30	5.43 (0.88**–**33.63)	0.069						
Hap0/Hap4	13	6.60	1	1.40	0.25 (0.03**–**2.11)	0.201	NA	NA	NA	NA	NA	NA						
Hap0/Hap5	**7**	**3.60**	**5**	**7.20**	**5.09 (1.23–21.03)**	**0.025**	1	0.50	1	1.40	2.66 (0.15**–**46.84)	0.505						

*ERCC8*	C. Breast and prostate cancer cohort consortium (BPC3)																	
	*ERCC6*																	
	Hap1/Hap1						Hap5/Hap1											
	Control		Case		Adjusted OR (95% CI)	p-Value	Control		Case		Adjusted OR (95% CI)	p-value						
	n	%	n	%			n	%	n	%								
Hap1/Hap1	55	25.82	50	27.78	1		**29**	**13.62**	**9**	**5.00**	**0.33 (0.12–0.88)**	**0.027**						
Hap2/Hap1	53	24.88	63	35.00	1.45 (0.77–2.71)	0.248	24	11.27	14	7.78	0.51 (0.21–1.23)	0.136						
Hap2/Hap2	22	10.33	12	6.67	0.52 (0.20–1.40)	0.197	7	3.29	13	7.22	1.64 (0.53–5.08)	0.391						
Hap1/Hap3	5	2.35	5	2.78	1.28 (0.31–5.31)	0.732	7	3.29	4	2.22	0.16 (0.02–1.42)	0.100						
Hap1/Hap4	8	3.76	7	3.89	1.25 (0.30–5.16)	0.754	3	1.41	3	1.67	0.65 (0.10–4.34)	0.660						

CGEMS: p-value for interaction = 0.010 (Wald Test, Chi-Square 19.98).

WHI: p-value for interaction = 0.034 (Wald Test, Chi-Square 31.66).

BPC3: p-value interaction = 0.047 (Wald Test, Chi-Square 21.21).

*NA* Not analyzed due to missing diplotypes or subjects (i.e., cell containing 0 subjects).

^A^Unconditional logistic regression analysis adjusted for family history of breast cancer.

^B^Unconditional logistic regression adjusted for family history of breast cancer, parity, oral contraceptive use, number of months of breast feeding, and body mass index.

^C^Unconditional logistic regression models adjusted for family history of breast cancer and consent group [i.e., three cohorts that make up our BPC3 dataset, namely Prostate, Lung, and Colorectal Cancer (PLCO), European Prospective Investigation into Cancer and Nutrition (EPIC), and Polish Breast Cancer Study (PBCS) and restricted to Caucasian ≥ 50 years of age.

#### National Surgical Adjuvant Breast and Bowel Project (NSABP)

The study design and subject characteristics of the NSABP^13,14^ dataset used in our study (430 cases, 822 controls) are described in the Methods section. All *ERCC6* and *ERCC8* SNPs analyzed in our NSABP dataset were in HWE and in strong LD among the controls (Supplementary Fig. 1C,D).

One *ERCC6* SNP, rs2229760 (NC_000010.11:g.49472987G > A) was associated with an increased risk for breast cancer among heterozygotes (OR 1.40, 95% CI 1.08–1.83, p = 0.010) (Table 1). No statistically-significant increased risks were found with any *ERCC8* SNPs or *ERCC6* and *ERCC8* Haplotypes and diplotypes (Supplementary Table 3). There were no significant interactions detected between *ERCC6* and *ERCC8* diplotypes in NSABP.

#### Women’s Health Initiative (WHI): Hormone herapy Trials

The study design and subject characteristics of the original WHI dataset^15,16^ and the subset dataset used in our study (465 cases and 1394 controls)^12^ have been published and are described in the Methods section. All *ERCC6* and *ERCC8* SNPs typed on these subjects were in HWE and in strong LD among the controls (Supplementary Fig. 1E,F). The most common haplotypes of *ERCC6* and *ERCC8* among the controls are also depicted in Supplementary Fig. 1E,F.

Statistically significant increased risk of breast cancer in WHI was found with heterozygotes at *ERCC6* rs1012553 (NC_000010.11:g.49532097A > T) (OR 1.35, 95% CI 1.07–1.71, p = 0.01) and rs2228528 (NC_000010.11:g.49524234C > T) (OR 1.29, 95% CI 1.01–1.66, p = 0.04) (Table 1). Haplotype analysis revealed statistically-significant increased risk of breast cancer with *ERCC6* Hap 2 (OR 1.36, 95% CI 1.03–1.78, p = 0.03) (Supplementary Table 4). Diplotype analysis revealed statistically-significant increased risk of breast cancer with diplotype 2/4 (OR 2.48, 95% CI 1.11–5.55, p = 0.03) (Supplementary Table 4).

None of the *ERCC8* SNPs, haplotypes or diplotypes were associated with an increased risk of breast cancer at a statistically-significant level. Joint effect analysis revealed increased risk of breast cancer with the *ERCC8* 0/0 and *ERCC6* 2/4 diplotype combination (OR 5.31, 95% CI 1.22–23.09, p = 0.026) and the *ERCC8* 0/5 and *ERCC6* 0/0 diplotype combination (OR 5.09, 95% CI 1.23, 21.03, p = 0.025) compared with the reference category (Table 2B). Gene–gene interaction analysis revealed statistically-significant interaction between *ERCC6* and *ERCC8* at the diplotype level (p_interaction_ = 0.034) (Table 2B).

#### Breast and Prostate Cancer Cohort Consortium (BPC3)

The demographic characteristics of subjects in the BPC3^17,18^ dataset used in our study (977 cases, 1026 controls) are described in the Methods section. All *ERCC6* and *ERCC8* SNPs typed on these subjects were in HWE and in strong LD among the controls (Supplementary Fig. 1G,H). The most common haplotypes for *ERCC6* (Supplementary Fig. 1G) and *ERCC8* (Supplementary Fig. 1H) among the controls are also depicted.

One *ERCC6* SNP, rs3750751, was associated with a significantly increased risk (OR 1.35, 95% CI 1.01–1.80, p = 0.04) of breast cancer among heterozygotes in BPC3 (Table 1), and the trend (i.e., increasing number of variant alleles) was also significant (OR 1.38, 95% CI 1.05–1.81, p = 0.02). This SNP was associated with an even higher risk of breast cancer (OR 1.88, 95% CI 1.17–3.02, p = 0.009) in stratified analysis in one of the three cohorts that made up our BPC3 dataset, namely the Polish Breast Cancer Study (PBCS) (Table 3). Haplotype and diplotype analysis did not reveal any statistically-significant associations in the entire BPC3 dataset; however, when stratified by cohort, significant association of *ERCC6* Hap 6 (OR 1.65, 95% CI 1.02–2.68, p = 0.04) with breast cancer was detected in PBCS (results not shown).

**Table 3:** Stratified analysis of *ERCC6* rs3750751 in the three cohorts of our Breast and Prostate Cancer Consortium (BPC3) dataset, namely Prostate, Lung, and olorectal Cancer (PLCO), European Prospective Investigation into Cancer and Nutrition (EPIC), and Polish Breast Cancer Study (PBCS).

Study	SNP rs3750751	Alleles	Case (N = 255)	Case %	Control (N = 340)	Control %	OR (95% CI)	p-value
PLCO		GG	211	82.75	283	83.24	1	
		GA	40	15.70	55	16.18	1.04 (0.66**–**1.63)	0.87
		AA	4	1.57	2	0.59	2.78 (0.50**–**15.39)	0.25
EPIC			**Case (N = 368)**	**Case %**	**Control (N = 354)**	**Control %**		
		GG	303	82.34	294	83.05	1	
		GA	65	17.66	58	16.38	1.25 (0.64**–**2.45)	0.51
		AA	0	0.00	2	0.56	NA	NA
PBCS			**Case (N = 354)**	Case %	**Control (N = 332)**	**Control %**		
		GG	298	84.18	302	90.96	1	
		GA	56	15.82	30	9.04	1.88 (1.17–3.02)	0.009
		AA	0	0.00	0	0.00	NA	NA

Unconditional logistic regression models adjusted for family history of breast cancer and restricted to Caucasian subjects ≥ 50 years.

*NA* Not analyzed due to small number of subjects (cells with 0 value).

None of *ERCC8* SNPs, haplotypes or diplotypes were associated with a statistically-significant increased risk of breast cancer (Supplementary Table 5). Gene–gene interaction analysis revealed borderline-significant interaction between *ERCC6* and *ERCC8* at the diplotype level (p_interaction_ = 0.047) (Table 2C).

### Analysis of gene-expression microarray datasets

**Figure 2:**
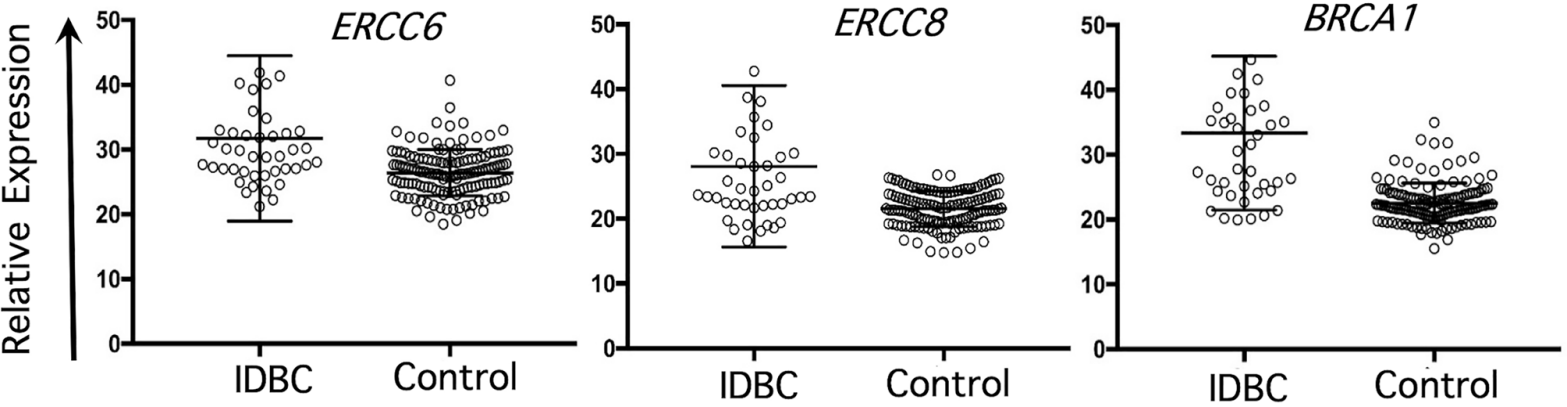
Individual analysis of gene expression microarray dataset GSE10780 containing invasive ductal breast cancer (IDBC) cases (n = 42) and control samples (n = 143) among peri- and post-menopausal women.

Individual analysis of raw data from two gene expression microarray datasets, one containing invasive ductal breast cancers (IDBC) and normal adjacent tissue, GSE10780^19^, and one from a case–control study of IDBC, E-TAMB-276^20^, revealed significant upregulation of *ERCC6* and *ERCC8*. In GSE10780, upregulation of *ERCC6* (fold change = 1.2, p = 1.5 × 10^–5^) and *ERCC8* (fold change = 1.3, p = 1.8 × 10^–8^) was comparable to that of *BRCA1* (fold change = 1.5, p = 1 × 10^–15^) (Fig. 2). Meta-analysis of GSE10780^15,16^ and E-TABM 276^15,16^ also revealed significant upregulation of *ERCC6* (p = 7.95 × 10^–6^) and *ERCC8* (p = 4.67 × 10^–6^).

### Analysis of TCGA dataset

**Figure 3:**
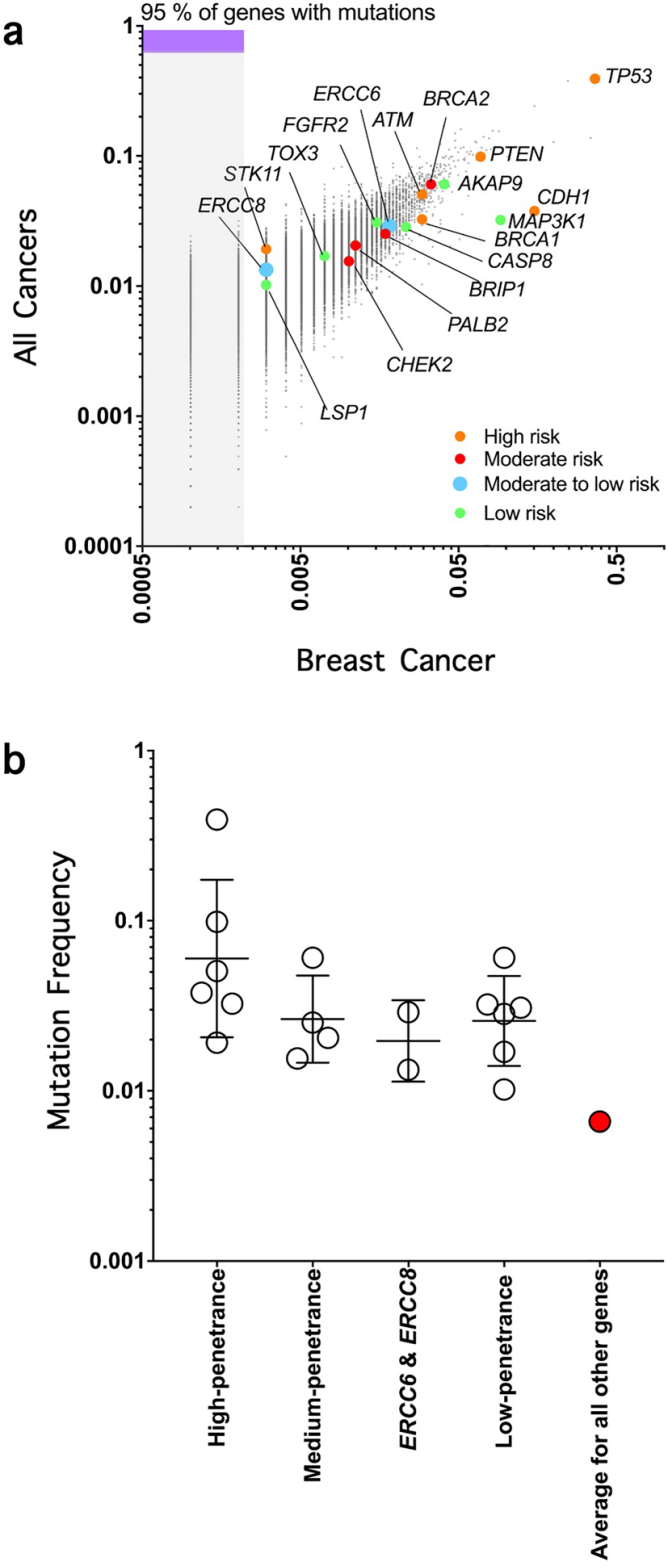
(**a**,**b**) Frequency of somatic mutations in *ERCC6* and *ERCC8* in comparison with known breast cancer susceptibility genes in the Cancer Genome Atlas (TCGA) Dataset. (**a**) Mutation frequency in all cancers versus breast cancer. (**b**) Mutation frequencies of breast cancer susceptibility genes in TCGA breast cancers.

Results of our analysis of TCGA^21^ (https://portal.gdc.cancer.gov/) breast tumor data revealed that 95% of genes mutated in breast tumors had mutation frequencies that were below 1% (Fig. 3a). All known breast cancer susceptibility genes, however, had mutation frequencies which were greater than 1%. We found that the mutation frequency of *ERCC6* (1.8%) in breast tumors was similar to those of known breast cancer susceptibility genes, such as *BRCA1* (2.9%), *BRCA2* (2.9%), *BLM* (1.9%), *FGFR2* (1.5%), and *CHEK2* (1.0%) (Fig. 3a). The mutation frequency of *ERCC8* (0.3%) in breast tumors was also similar to some of the other known breast cancer susceptibility genes such as *STK11* (0.3%) and *LSP1* (0.3%) (Fig. 3a).

Using TCGA breast tumor data, we compared mutation frequencies of *ERCC6* and *ERCC8* to those of breast cancer susceptibility genes with high-penetrance (*BRCA1*, *BRCA2*, *TP53*, *PTEN*, *CDH1* and *STK11*), medium-penetrance (*BLM*, *ATM*, *CHEK2, BRIP1,* and *PALB2*), and low-penetrance (*FGFR2*, *MAP3K1*, *CASP8, TOX3,* and *LSP1*), and to the rest of the genome. This analysis revealed that high-penetrance breast cancer susceptibility genes had higher mutation frequencies than medium- and low-penetrance genes (Fig. 3b), which had similar mutation frequencies to each other and to *ERCC6* and *ERCC8* (Fig. 3b). Analysis of TCGA breast tumors also revealed a higher mutation frequency for all known breast cancer susceptibility genes and for *ERCC6* and *ERCC8* compared to the average for all other genes (Fig. 3b).

**Figure 4:**
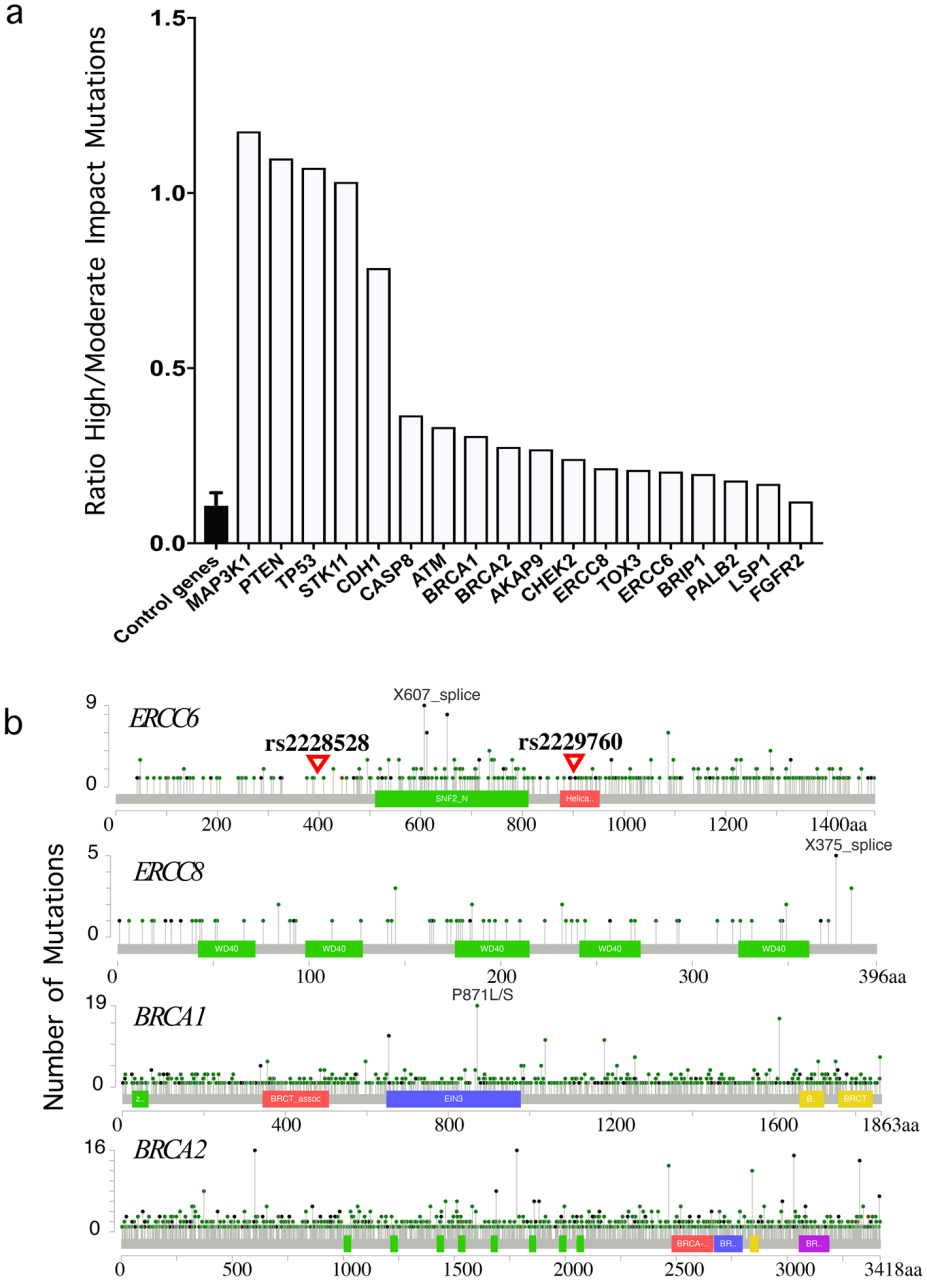
(**a**,**b**) Mutation analysis of selected genes in all cancers in the Cancer Genome Atlas (TCGA) dataset. (**a**) Ratio of high- to moderate-impact mutations in *ERCC6* and *ERCC8* in comparison to known breast cancer susceptibility genes and control genes. (**b**) Somatic mutation landscape of *ERCC6*, *ERCC8*, *BRCA1* and *BRCA2.*

Using data on all cancers in TCGA, we compared the ratio of high-impact mutations (stop-gained, frame-shift variant, splice-acceptor variant, splice-donor variant, start-lost, stop-lost) to moderate-impact mutations (missense variant, in-frame deletion, in-frame insertion, protein-altering variant, splice-region variant, incomplete terminal codon variant) in *ERCC6* and *ERCC8* versus the known high-, medium- and low-penetrance breast cancer susceptibility genes versus a group of size-matched (i.e., matched based on gene length) control genes (i.e., genes not involved in breast cancer susceptibility, which included those coding for immunoglobulin and T-cell receptors as well as olfactory receptors). We identified 200 moderate-impact and 41 high-impact mutations in *ERCC6* and similar numbers in *ERCC8*. The ratios of high- to moderate-impact mutations for *ERCC6* (0.20) and *ERCC8* (0.22) were similar to those for *BRCA1* (0.30), *BRCA2* (0.30), *CHEK2* (0.28), and *BLM* (0.20) (Fig. 4a). Somatic mutation landscape of *ERCC6*, *ERCC8*, and the *BRCA* genes are depicted in Fig. 4b (obtained from https://www.cbioportal.org/), and show the location of two of our significant *ERCC6* SNPs located in exons, rs2228528 and rs2229760, with respect to the reported mutations in TCGA.

## Discussion

Using a hypothesis-driven (candidate-gene) integrative genetic epidemiologic approach to analysis of raw multi-omics data, we tested the association of *ERCC6* and *ERCC8* with peri- and post-menopausal breast cancer. Our candidate-gene association study involving individual analysis of four GWAS datasets (containing total of 2882 cases and 4397 controls) found 30% to threefold increased risk of breast cancer (OR 1.30 with rs2228528 in WHI to OR 2.91 with rs3750751 in CGEMS) conferred by six *ERCC6* variations. The MAF of these six *ERCC6* variations ranged from 11.8 for rs3750751 to 40.9% for rs4838519 in the general Caucasian population [based on MAF reported in the NCBI (https://www.ncbi.nlm.nih.gov/snp/)].

Our findings were consistent at the SNP, haplotype and diplotype level within each dataset. In CGEMS, the associated *ERCC6* haplotype, Hap 5, contained the variant alleles at two of the associated SNPs, rs3750749 and rs4253082. Furthermore, Hap 5 was one of the haplotype pairs in diplotype 2/5, which was associated with nearly threefold increase in risk. One of the other *ERCC6* diplotypes associated with nearly threefold increase in risk in CGEMS, 4/4, contained the variant allele at the third associated SNP, rs375075. Similarly, the associated haplotype in the WHI, Hap 2, contained the variant alleles at the two associated SNPs, namely rs2228528 and rs1012553; Hap 2 was one of the haplotype pairs in diplotype 2/4, which was associated with 2.5-fold increased risk in this dataset. In BPC3, Hap 6 was the only haplotype associated with a borderline significant increased risk and was also the only common haplotype that contained the variant allele at the associated SNP, rs3750751.

The results of joint effect analyses were also compatible with main effect analyses within each dataset in that *ERCC6* diplotypes with main effects were those highlighted in joint effects analyses. For example, in CGEMS, joint effect analysis highlighted diplotype 2/5, which had a significant main effect in diplotype analysis. Similarly, in WHI, joint effect analysis highlighted diplotype 2/4. Even though it is difficult to compare associated haplotypes and diplotypes across different datasets (due to different genotyping platforms and different SNPs typed in each dataset as well as LD between typed and untyped SNPs), consistency can also be noted across the analyzed datasets. One *ERCC6* SNP (rs3750751) was significant in 2 of 3 GWAS where it was typed (this SNP is discussed in detail below).

In order to gain additional support for our findings, we conducted analyses of transcriptome and TCGA data. In general, somatic transcript levels and mutation frequencies do not necessarily parallel germline mutation profiles. Therefore, our findings from these analyses, although consistent with the results of our analyses of GWAS datasets, should be interpreted only as additional evidence. Analysis of transcriptome datasets showed statistically- significant upregulation of *ERCC6* and *ERCC8* in breast cancer, which is notable given the large sample size (total of 65 breast cancer samples and 153 normal tissue samples in the meta-analysis and 42 breast cancer samples and 143 normal tissue control samples in GSE10780 in individual analysis). The similarity of ERCC6 (and ERCC8) to BRCA1 in terms of both expression level and fold-change estimate is also noteworthy. BRCA1 levels are normally elevated in tumor biopsies from breast cancer patients who do not carry a germline mutation in the *BRCA1*^22^. This is beleived to be due to the involvement of BRCA1 in cell cycle control and DNA repair processes^23^.

We also assessed protein expression levels of ERCC6 and ERCC8 in breast cancer using the Broad Institute proteome database (https://prot-shiny-vm.broadinstitute.org:3838/CPTAC-BRCA2016/) and found elevated expression of both, similar to some of our other comparison proteins such as CHEK2 (Data not shown).

Analysis of 522 TCGA breast cancers (with mutation information on 17,243 genes) also provided supporting evidence for involvement of *ERCC6* as it revealed similar frequency of mutations and similar ratio of high- to low-impact mutations in *ERCC6* and *ERCC8* compared to known breast cancer susceptibility genes such as *BRCA1*, *BRCA2*, *CHEK2*, and *BLM*.

Although, the main effects of *ERCC8* variations on breast cancer risk did not reach statistical significance in some of our GWAS datasets, we cannot rule out a main or modifying effect for *ERCC8* given our findings of possible interaction between *ERCC6* and *ERCC8* in three of four GWAS analyzed. The findings from our analyses of transcriptome and TCGA datasets further suggest a potential main or modifying effect for *ERCC8* in breast cancer susceptibility.

One *ERCC6* SNP (rs3750751) was significant in 2 of 3 GWAS where it was typed. While heterozygotes at this SNP had 35% increased risk in BPC3 (and 88% in PBCS, 1 of 3 cohorts pulled together to form our BPC3 dataset), homozygotes for the variant allele at rs3750751 had close to threefold increased risk of breast cancer in CGEMS. The frequency of heterozygotes at *ERCC6* rs3750751 ranged from 14 to 16% among the controls in our GWAS datasets. The homozygote variant frequency for this SNP was less than 1% among controls in our datasets. The minor allele frequency (MAF) of rs3750751 was 7.2–8.6% among the controls in our datasets (Supplementary Table 1) and has been reported as 11.8% in the general Caucasian US population (https://www.ncbi.nlm.nih.gov/snp/).

*ERCC6* rs3750751 has not been previously implicated in breast cancer risk, however it has been reported to be associated with an increased risk of bladder cancer in one study^24^. rs3750751 lies in the 3′ untranslated region (3′-UTR), which has been proposed to be involved in post-transcription regulation of protein expression^25^. Recent studies implicate the role of micro RNA (miRNA)s in such regulation, suggesting that SNP variants located in 3′ UTRs may destroy or create miRNA binding sites^26^, thus influencing tumor susceptibility^27^. Each miRNA may be able to repress hundreds of gene targets post-transcriptionally, therefore, they are powerful regulators of gene expression^28^. miRNAs are involved in regulating a diverse set of biological processes including growth, differentiation and apoptosis^29^. The other *ERCC6* variations associated with breast cancer in our study were rs3750749, rs4253082, and rs1012553, all located in the introns, and rs2228528 and rs2229760, which are missense variants in the exons. An interaction with smoking (pack-years) was noted for rs4253082 in one bladder cancer study^30^ and a possible association of rs2228528 with muscle-invasive bladder tumors was noted in another study^31^. To establish the role of these SNPs in cancer susceptibility and elucidate the underlying mechanism of carcinogenesis, functional assays are needed.

Analysis of different breast cancer GWAS to date have led to associations with 182 variants^2,10,11,32–43^, a few of which have been validated. One validated low-penetrance breast cancer susceptibility gene, *FGFR2*^10^, has been found to be associated with 26% increased risk of breast cancer (OR 1.26, 95% CI 1.23–1.30) and has a MAF of > 30% in the general population. In comparison, our findings suggest 30% to threefold increased risk of breast cancer for *ERCC6* variations with MAF of 11.8–40.9% in the general population. Lack of identification of *ERCC6* in previous studies may be due to inherent limitations of GWAS and candidate gene studies. A major limitation of GWAS is that they are agnostic and exploratory, hence the high probability of false positive associations and the need for very large sample sizes for detection of weak main effects and gene–gene interactions. To alleviate the multiple-testing large false positive burden, stringent threshold levels for significance (i.e., p < 0.0001 for main effects and p < 10^–8^ for interactions) have been recommended for GWAS. This stringent threshold levels may lead to many true positive associations being missed. Candidate-gene approaches, which study association between variations in a small number of genes with the risk of disease, are more powerful since they are hypothesis-driven and have a lower probability of false-positive associations (hence, may be guided by conventional significance threshold levels). However, selection of suitable candidate genes is challenging and may be imprecise.

Selection of *ERCC6* and *ERCC8* as candidates for our integrative genomic study reported here was based on our previous genetic epidemiologic studies of DNA repair disorders^44–46^, which led to the hypothesis that genes involved in the nucleotide excision repair (NER) pathway may be involved in breast cancer susceptibility. *ERCC6* and *ERCC8* code for the main components of the transcription-coupled (TC) repair sub-pathway of the NER, which repairs damage to actively-transcribed regions of DNA^47,48^ caused by ultraviolet radiation, chemicals and free radicals^49^. Free radicals and reactive oxygen species (ROS) are associated with oxidative stress, a mechanism relevant to cancer. It has been observed that TC-NER is induced as the result of damage caused by oxidative stress, and that ERCC6 accumulates at sites of locally induced oxidative damage in vivo in a transcription-dependent manner^50^. It has also been observed that cells with mutations in *ERCC6* and *ERCC8* are sensitive to ROS^51^. The effect of *ERCC6* and *ERCC8* variants analyzed in our study on TC-NER is not known. However, given that TC-NER is a critical protective pathway against genotoxic agents^52^, it is plausible that certain variations in *ERCC6* and *ERCC8* may affect protein function, and repair efficiency of TC-NER, hence increase susceptibility to cancer.

In our study, we found some evidence for interaction between *ERCC6* and *ERCC8* diplotypes. Our findings of interaction are biologically plausible as these genes work together in the same biological pathway and sub-pathways. Our results suggest that TC-NER and global genome (GG)-NER may be good candidates for future pathway-based association studies of breast cancer.

The public health significance of this study stems from its potential to provide leads for inclusion of additional low- to moderate-risk breast cancer susceptibility genes (upon replication and proper validation in clinical studies) in the panel of susceptibility genes for risk scores^41,53^. Currently, genetic screening and counseling regarding personalized preventive and management strategies (such as increased surveillance, chemoprevention and prophylactic surgery) are available to individuals from families and ethnic groups with identified high- and medium-penetrance mutations^3^. Multigene panel tests for breast cancer exist and are offered to women who meet the criteria for clinical genetic testing such as early age of onset, bilateral and/or triple-negative disease, and a family history^54^. In theory, incorporation of additional low- and moderate-risk genes into multigene panel tests, following substantial validation and efficacy studies, may improve prevention strategies.

Strengths of our study include its unique integrative nature and uniform application of inclusion/exclusion factors to all GWAS and transcriptome datasets. One limitation of our study, which was related to the secondary nature of our datasets, was lack of information on individual estrogen receptor (ER) and progesterone receptor (PR) status in all datasets and inclusion of only ER-negative cases in BPC3. Stratifying by receptor status may modify the associations observed in our study or identify new associations. Nevertheless, the fact that our findings with respect to *ERCC6* associations were consistent in all four datasets despite the differences in ER and PR status of the subjects further strengthens our results.

Another limitation of our study (also due to the secondary nature of our datasets) was lack of information on all risk-modifying environmental and reproductive/behavioral variables. We were limited by the variables which were provided to us as part of each dataset. While we had information on nearly all confounders in WHI, we were only provided with 5-year age categories and family history information in CGEMS. Despite this limitation, we utilized all information provided to us on all reproductive and behavioral variables in order to properly adjust our odds ratios for the effect of confounders. Our future studies to replicate and confirm these findings would need to involve primary data collection of pre-, peri- and post-menopausal breast cancer cases. Future studies would also need to validate and extend our findings to other populations and racial/ethnic groups.

## Conclusions

Using a hypothesis-driven integrative genetic epidemiologic approach to analysis of multi-omics data, we propose that *ERCC6* may be a previously-unreported low- to moderate-risk breast cancer susceptibility gene, and that it may interact with *ERCC8*. Our results suggest that NER may be a good candidate for future pathway-based association studies of breast cancer. Our findings have the potential to provide a deeper insight into the genetic basis of common breast cancer and, following proper replication and validation, generate leads for improved prevention methods.

## Materials and methods

### Candidate gene and single nucleotide polymorphism (SNP) selection

Based on our previous genetic epidemiologic studies of DNA repair disorders^44–46^, *ERCC6* and *ERCC8*^52^*,* were selected as part of a panel of candidate genes for association studies with breast cancer. All SNPs within *ERCC6* and *ERCC8* (including those in the upstream and downstream regulatory regions) which were typed in selected GWAS datasets (described below) in at least 80% of the subjects were used for analysis. After quality control exclusions, our roster of SNPs within the two candidate genes included 12 *ERCC6* and 2 *ERCC8* SNPs in CGEMS, 17 *ERCC6* and 3 *ERCC8* SNPs in NSABP, 14 *ERCC6* and 8 *ERCC8* SNPs in WHI, and 16 *ERCC6* and 3 *ERCC8* SNPs in BPC3. Information about these SNPs along with their chromosomal positions [obtained from the reference sequence in the National Center for Biotechnology Information (NCBI) (https://www.ncbi.nlm.nih.gov/snp/) Build 38.p12] and physical locations within the gene are listed in Supplementary Table 1.

### Selection and statistical analysis of genome-wide association studies (GWAS) datasets

We selected case–control GWAS datasets which contained cases with invasive ductal breast cancer (IDBC) diagnosed peri- or post-menopause (≥ 50 years of age) and healthy controls (with no personal history of breast cancer) appropriately-matched to the cases based on a number of criteria by the original investigators. Race was restricted to Caucasian to avoid population stratification^55^.

Raw data from four GWAS datasets fitting our inclusion criteria were downloaded from the National Institutes of Health (NIH) after obtaining approvals from the corresponding data repository committees and stored on a computer and/or the server in RM’s laboratory at the University at Albany. The datasets analyzed in this study included the Cancer Genetic Markers of Susceptibility (CGEMS)^10,11^ (1089 cases, 1093 controls), a nested case–control (430 cases, 822 controls) within the National Surgical Adjuvant Breast and Bowel Project (NSABP)^13,14^, a nested case–control (465 cases and 1394 controls) within the Women's Health Initiative (WHI)^15,16^ Hormone Therapy Trials, and a nested case–control (977 cases, 1026 controls) within the Breast and Prostate Cancer Cohort Consortium (BPC3)^17,18^. Institutional Review Board (IRB) approval was obtained from University at Albany [by RM] for analyses of secondary datasets (Protocol #11-298 and #11-E-177) reported in this paper. Detailed information about each secondary dataset is provided below.

Raw data from CGEMS^10,11^, a case–control study nested within the Nurse’s Health Study (NHS) cohort containing genotype information at 528,178 single nucleotide polymorphism (SNP) loci on 1145 post-menopausal women of European ancestry with invasive breast cancer and 1,142 controls was downloaded from the NIH [accession #6175-13 for version phs000147/GRU]. Detailed information about the design and original analyses of CGEMS data is available from published reports^33^. For this dataset, we restricted the analysis to cases diagnosed at ≥ 55 years of age (N = 1089) and frequency-matched controls (N = 1093). Besides 5-year age categories, the only other phenotype variable available to us on these subjects was presence or absence of a first-degree family history of breast cancer.

Raw data from a nested case–control study within the PGRN-RIKEN Mayo National Surgical Adjuvant Breast and Bowel Project (NSABP) Prevention Trials (P-1^14^ and P-2^13^) were downloaded from the NIH (accession #10821-11 for version phs000305/APTC). This dataset contained genotype information at 601,273 SNPs on 592 breast cancer cases and 1171 controls of European (Caucasian) ancestry. We restricted our analysis to women diagnosed with breast cancer ≥ 50 years of age (N = 430) and appropriately-matched controls (N = 822). In NSABP^13,14^, controls were matched to cases based on several factors by the original study investigators; the matching factors included age at trial entry, time in the study, history of lobular carcinoma in situ, and 5-year predicted breast cancer risk based on the Gail model.

Raw data from the Women's Health Initiative (WHI)^15,16^ Hormone Therapy Trials containing genotype information at 1,051,295 SNPs [in Genomics and Randomized Trials Network (GARNET) subgroup of WHI] and 733,202 SNPs [in Women’s Health Initiative Memory Study (WHIMS) group] on a total of 10,634 subjects (4894 in GARNET and 5740 in WHIMS) was downloaded [NIH accession #11295-10 for version phs000200/HMB-IRB and #11296-10 for phs000200/HMB-IRB-NPU]. We created a nested case–control dataset of women diagnosed with invasive breast cancer ≥ 50 years of age (N = 465) and healthy controls (N = 1394) frequency-matched to the cases based on age in 3:1 control to case ratio. Variables provided to us on WHI subjects included a number of confounders of breast cancer risk such as family history of breast cancer, parity, oral contraceptive use, breast feeding, and body mass index, which we adjusted for in our analyses. The demographic characteristics of the 465 cases in our study and the entire control population that gave rise to the 1394 controls included in our analyses were published in our previous report^12^.

Raw data from the Breast and Prostate Cancer Cohort Consortium (BPC3)^17,18^ was downloaded from the NIH [accession #40019-3 for version phs000812/HMB-PUB-MDS, #40020-3 for phs000812/CADM, and #40021-3 for phs000812/DS-BOED-MDS]. The parent BPC3 GWAS included cases and controls from eight studies, but the subset BPC3 dataset which was made available to us contained genotype information at 550,000 loci on 1309 ER-negative breast cancer cases and 1351 appropriately-matched controls from three of the original BPC3 cohorts, namely the Prostate, Lung, Colorectal, and Ovarian (PLCO) Cancer Screening Trial, the European Prospective Investigation into Cancer and Nutrition (EPIC), and the Polish Breast Cancer Study (PBCS). We restricted our analysis to breast cancer cases diagnosed ≥ 50 years of age (N = 977) and appropriately-matched healthy controls (N = 1026) in this subset BPC3-PLCO/EPIC/PBCS dataset, referred to as BPC3 in this paper. Phenotype variables provided to us in this dataset included family history of breast cancer, which was adjusted for in our analyses.

For each gene, linkage disequilibrium (LD) patterns and deviations from Hardy–Weinberg Equilibrium (HWE) were assessed among the controls in each dataset using Haploview^56^ and a χ^2^ test of independence. Individual haplotypes and diplotypes (haplotype pairs) were determined for all subjects using PHASE^57,57^. Logistic regression models were used to calculate odd ratios (OR) and 95% confidence intervals (CI) for association between breast cancer and each SNP, haplotype and diplotype, while adjusting for covariates. Unconditional logistic regression was used for analyses of CGEMS, WHI and BPC3 data, while conditional logistic regression was used to analyze NSABP. The CGEMS, NSABP, and WHI datasets contained combination of both estrogen receptor (ER)-positive and -negative cases where as the BPC3 dataset made accessible to us contained ER-negative cases only. All datasets contained combination of progesterone (PR)-positive and –negative cases. The individual ER and PR status of cases were not made available to us for any of the GWAS datasets. Joint effects of *ERCC6* and *ERCC8* diplotypes on risk of breast cancer were examined and multiplicative interaction at the diplotype level was tested by assessing statistical significance of the interaction terms in the logistic regression models using the Wald test. All association testing was done using SAS version 9.3 (SAS Institute, Cary, NC) and SPSS version 24 (IBM SPSS Statistics).

### Selection and statistical analysis of gene expression microarray datasets

Raw data from two gene expression microarray datasets, GSE10780^19^ and E-TABM 276^20^, containing normal and cancerous breast tissue, were downloaded from Gene Expression Omnibus (GEO) and Array Express, respectively, and used for meta-analysis. As per the inclusion criteria, both datasets ascertained cases of IDBC among peri- and post-menopausal Caucasian women. Control samples in GSE10780^19^ contained normal adjacent tissue from breast cancer cases. Controls in E-TABM 276^20^ had no personal history of breast cancer and contributed histologically normal breast tissue. Other inclusion criteria were presence of more than five samples per group separated by more than two standard deviations (SD) difference between the groups in Principle Component Analysis (PCA) using first two axes of the PCA. GSE10780 contained 42 IDBC cases and 143 control samples and E-TAMB-276 contained 23 IDBC cases and 10 healthy controls.

The data was geometric-mean normalized and analyzed using non-parametric T-test. Meta-analysis was conducted on GSE10780^19^ and E-TABM-276^20^ by calculating the p-values for each gene in each experiment using student’s t-test. P-values for genes with discordant fold differences between datasets were changed to “one” and Fisher’s method was used to calculate meta-analysis p-values. Fold changes were averaged. All statistical analyses of gene expression microarray data were done using Partek Genomics version 6.6 (Partek Inc., St. Louis, MO, USA).

### Statistical analysis of the cancer genome atlas (TCGA) dataset

We sought to validate our findings by examining frequency of somatic mutations in The Cancer Genome Atlas (TCGA)^21^. We analyzed TCGA breast tumor data by comparing frequency of mutations in *ERCC6* and *ERCC8* with the frequencies in known breast cancer susceptibility genes and the rest of the genome. We also compared the ratio of high-impact mutations (stop-gained, frame-shift variant, splice-acceptor variant, splice-donor variant, start-lost, stop-lost) to moderate-impact mutations (missense variant, in-frame deletion, in-frame insertion, protein-altering variant, splice-region variant, incomplete terminal codon variant) in *ERCC6* and *ERCC8* versus several comparison groups in the TCGA. One comparison group included all known high-, medium- and low-penetrance breast cancer susceptibility genes. Another comparison set included a group of size-matched (i.e., matched based on gene length) control genes (i.e., genes not involved in breast cancer susceptibility, which included those coding for immunoglobulin and T-cell receptors as well as olfactory receptors). All analyses of the TCGA data were done through the TCGA portal^21^ (https://portal.gdc.cancer.gov/).

Additionally, we created the somatic mutation landscape of *ERCC6*, *ERCC8*, and the *BRCA* genes using https://www.cbioportal.org/) and assessed protein expression levels of ERCC6 and ERCC8 in breast cancer using the Broad Institute proteome database (https://prot-shiny-vm.broadinstitute.org:3838/CPTAC-BRCA2016/).

## Supplementary information

Supplementary Information.

## Data Availability

All datasets analyzed in this study are available from the relevant NIH data repositories mentioned in the text as follows: Database for Genotypes and Phenotypes (dbGaP) (https://dbgap.ncbi.nlm.nih.gov/), GEO (https://www.ncbi.nlm.nih.gov/geo/), and TCGA (https://www.cancer.gov/tcga). All data analysis programs/software used in this study are outlined in the respective statistical analysis sub-sections of the “Materials and methods” and “Results” sections.
