# Supplementary material for: Integrative genomic analysis implicates ERCC6 and its interaction with ERCC8 in susceptibility to breast cancer
Source: Sci Rep. 2020 Dec 4;10:21276. doi: 10.1038/s41598-020-77037-7 (PMC7718875; doi:10.1038/s41598-020-77037-7)
Supplement: Supplementary file 1 — Supplementary Information. [file 41598_2020_77037_MOESM1_ESM.docx]

**Supplementary Figures and Tables**

**Integrative genomic analysis implicates *ERCC6* and its interaction with *ERCC8* in susceptibility to breast cancer**

**Roxana Moslehi^1*^, Hui-Shien Tsao^1,2^, Nur Zeinomar^1,3^, Cristy Stagnar^1,4^, Sean Fitzpatrick^1^, and Amiran Dzutsev^5^**

^1^ School of Public Health, University at Albany, State University of New York (SUNY), New York, USA

^2^ New York State Office of Children and Family Services, New York, USA

^3^ Mailman School of Public Health, Columbia University, New York, USA

^4^ Drukier Institute for Children’s Health, Weill Cornell Medicine, New York, USA

^5^ Cancer Vaccine Branch, National Cancer Institute, National Institutes of Health, Maryland, USA

* Corresponding author: Dr. Roxana Moslehi, School of Public Health, and Center for Excellence in Cancer Genomics, University at Albany, Albany, NY 12144, USA.

Tel: +1 518-402-0292; E-mail: [rmoslehi@albany.edu](mailto:rmoslehi@albany.edu)

**Supplementary Figure 1A-H. Linkage Disequilibrium (LD) Plots and Haplotype Frequencies of *ERCC6* and *ERCC8* in each Genome-Wide Association Study (GWAS) Dataset**

Linkage Disequilibrium (LD) Map: Sequence of single nucleotide polymorphisms (SNPs) along the top represents their relative order 5′ to 3′ (right to left) on the chromosome among the controls in each genome-wide association studies (GWAS) datatset. The red squares indicate a statistically-significant allelic association between the pair of SNPs. The thick line indicates SNPs in LD using the ‘solid spine of LD’ or ‘Confidence intervals (Gabriel et al.)’ algorithm in Haploview. White and pink squares indicate lack of LD between the SNPs. Numbers inside the square are the value of D′ multiplied by 100. D′=1 indicates complete LD while D′=0 indicates complete linkage equilibrium

Haplotype Map: The blue bar represents the most common allele and the pink bar represents the variant allele. Marker numbers are shown across the top for each gene. Frequencies are shown besides each haplotype for the entire pool of controls. Absence of haplotype maps signifies inability to reconstruct haplotypes due to small number of SNPs (i.e., ≤3) typed.

1. ***ERCC6* in Cancer Genetic Markers of Susceptibility (CGEMS)**


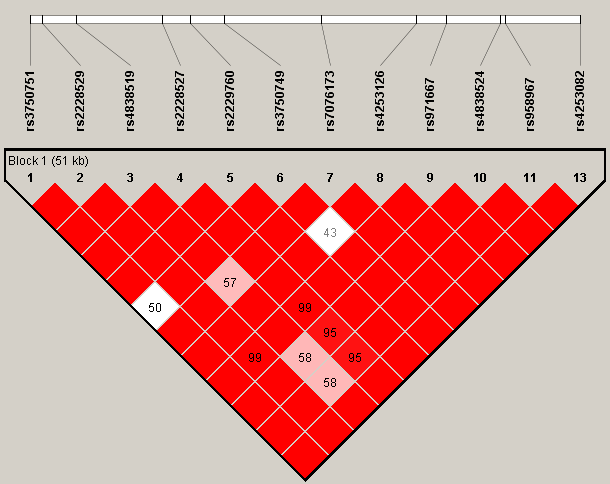

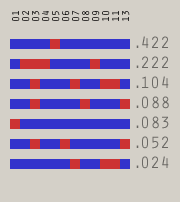


1. ***ERCC8* in CGEMS**


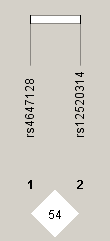


1. ***ERCC6* in National Surgical Adjuvant Breast and Bowel Project (NSABP)**

**
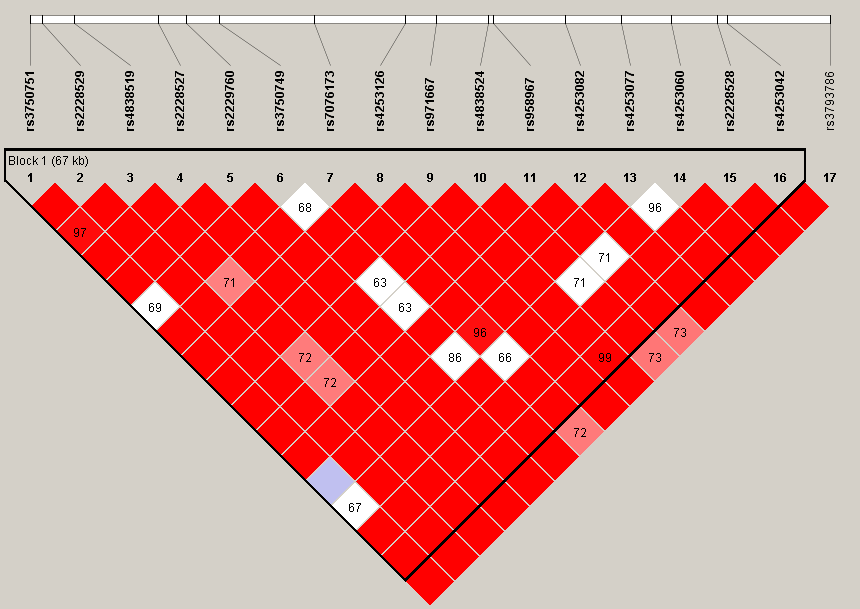

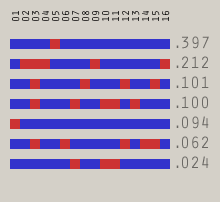
**

1. ***ERCC8* in NSABP**

**
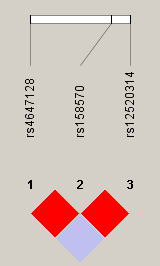
**

1. ***ERCC6* in Women’s Health Initiative (WHI) Hormone Therapy Trials**

**
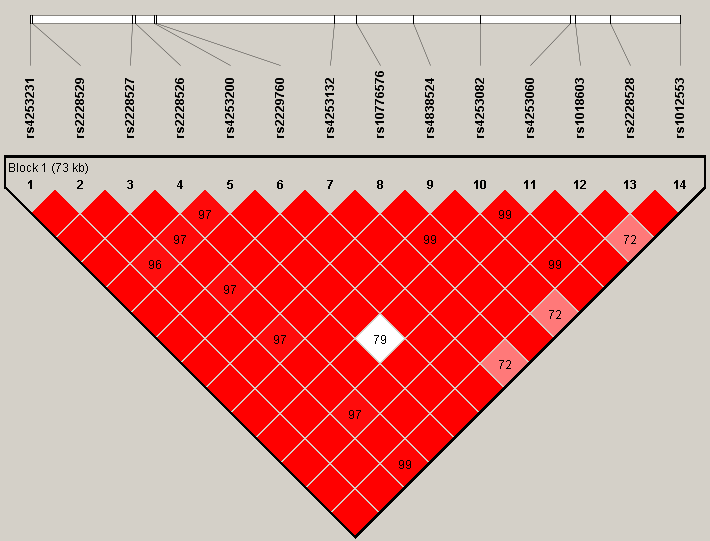

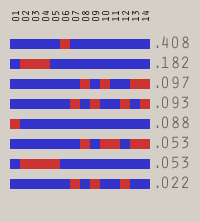
**

1. ***ERCC8* in WHI**


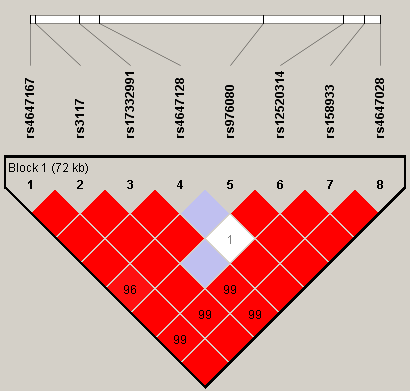

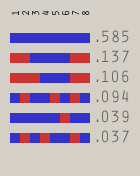


1. ***ERCC6* in Breast and Prostate Cancer Cohort Consortium (BPC3)**

**
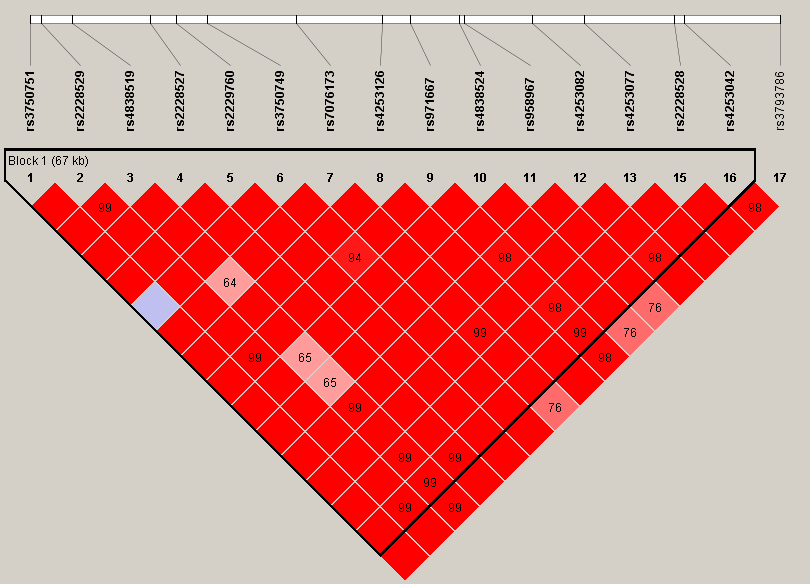

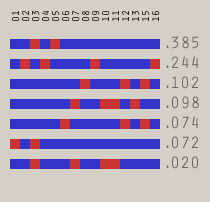
**

1. ***ERCC8* in BPC3**


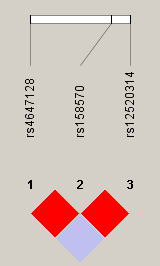


**Supplementary** **Table 1. Location and Minor Allele Frequency (MAF) of *ERCC6* and *ERCC8* Single Nucleotide Polymorphisms (SNPs) used in Our Study**

| **Gene (Chromosome)** | **SNP ID** | **Gene Location^a^** | **Position^a^** | **Common Allele^a^** | **Minor Allele Frequency (MAF) in GWAS Datasets and 1000(G)enomes^a^** | | | |  |
| --- | --- | --- | --- | --- | --- | --- | --- | --- | --- |
|  |  |  |  |  | **CGEMS^b^** | **NSABP^c^** | **WHI^d^** | **BPC3^e^** | **1000G^a^** |
| ***ERCC8*/*CSA* (5q12.1)** |  |  |  |  |  |  |  |  |  |
|  | **rs4647028** | Upstream | 60945999 | C |  |  | 0.243 |  | T: 0.140 |
|  | **rs158933** | Intron | 60942763 | A |  |  | 0.376 |  | A: 0.375 |
|  | **rs12520314** | Intron | 60938339 | T | 0.0429 | 0.0373 | 0.040 | 0.034 | C: 0.059 |
|  | **rs158570** | Intron | 60929087 | A |  | 0.3810 |  | 0.401 | C: 0.374 |
|  | **rs976080** | Intron | 60921824 | G |  |  | 0.094 |  | A: 0.138 |
|  | **rs4647128** | Intron | 60887717 | A | 0.0296 | 0.0413 | 0.038 | 0.044 | C: 0.017 |
|  | **rs17332991** | Intron | 60883533 | C |  |  | 0.107 |  | A: 0.043 |
|  | **rs3117** | 3'-UTR | 60874510 | T |  |  | 0.378 |  | G: 0.307 |
|  | **rs4647167** | 3'-UTR | 60873324 | A |  |  | 0.244 |  | C: 0.137 |
| ***ERCC6*/*CSB* (10q11.23)** |  |  |  |  |  |  |  |  |  |
|  | **rs3793786** | Intron | 49535042 | C |  | 0.2743 |  | 0.276 | G: 0.4093 |
|  | **rs1012553** | Intron | 49532097 | T |  |  | 0.245 |  | A: 0.409 |
|  | **rs4253042** | Intron | 49525181 | A |  | 0.2098 |  | 0.244 | C: 0.1647 |
|  | **rs2228528** | Exon | 49524234 | G |  | 0.1686 | 0.151 | 0.177 | A: 0.238 |
|  | **rs1018603** | Intron | 49520325 | A |  |  | 0.114 |  | C: 0.123 |
|  | **rs4253060** | Intron | 49519741 | G |  | 0.0680 | 0.054 | 0.075 | T: 0.176 |
|  | **rs4253077** | Intron | 49514923 | G |  | 0.1052 |  | 0.098 | A: 0.1068 |
|  | **rs4253082** | Intron | 49509540 | G | 0.1401 | 0.1691 | 0.151 | 0.178 | A: 0.297 |
|  | **rs958967** | Intron | 49502574 | A | 0.1302 | 0.1261 |  | 0.119 | G: 0.0996 |
|  | **rs4838524** | Intron | 49502077 | T | 0.1304 | 0.1261 | 0.115 | 0.118 | C: 0.100 |
|  | **rs971667** | Intron | 49497048 | G | 0.2223 | 0.2098 |  | 0.245 | A: 0.1821 |
|  | **rs10776576** | Intron | 49495587 | G |  |  | 0.15 |  | A: 0.223 |
|  | **rs4253126** | Intron | 49494144 | C | 0.0866 | 0.0997 |  | 0.102 | T: 0.047 |
|  | **rs4253132** | Intron | 49493110 | T |  |  | 0.115 |  | G: 0.123 |
|  | **rs7076173** | Intron | 49485304 | T | 0.1283 | 0.1233 |  | 0.118 | C: 0.0799 |
|  | **rs3750749** | Intron | 49476182 | T | 0.0517 | 0.0684 |  |  | C: 0.180 |
|  | **rs2229760** | Exon | 49472987 | G | 0.4219 | 0.4033 | 0.395 | 0.384 | A: 0.254 |
|  | **rs4253200** | Intron | 49472799 | A |  |  | 0.054 |  | C: 0.025 |
|  | **rs2228526** | Exon | 49470671 | A |  |  | 0.236 |  | C: 0.183 |
|  | **rs2228527** | Exon | 49470323 | A | 0.2236 | 0.2104 | 0.236 | 0.244 | C: 0.185 |
|  | **rs4838519** | Intron | 49462211 | A | 0.4693 | 0.4847 |  | 0.480 | C: 0.4093 |
|  | **rs2228529** | Exon | 49459059 | A | 0.2222 | 0.2106 | 0.235 | 0.241 | C: 0.179 |
|  | **rs4253231** | 3'-UTR | 49458762 | T |  |  | 0.088 |  | G: 0.141 |
|  | **rs3750751** | 3'-UTR | 49457882 | G | 0.0842 | 0.086 |  | 0.072 | A: 0.118 |

^a^<https://www.ncbi.nlm.nih.gov/snp/> (GRCh38.p12)

^b^Cancer Genetics Markers of Susceptibility (CGEMS)

^c^National Surgical Adjuvant Breast and Bowel Project (NSABP)

^d^Women’s Health Initiative (WHI) Hormone Therapy Trials

^e^Breast and Prostate Cancer Cohort Consortium (BPC3)

**Supplementary** **Table 2. Single Nucleotide Polymorphism (SNP), Haplotype, and Diplotype Analysis in Cancer**

**Genetic Markers of Susceptibility (CGEMS)**

| ***ERCC6* SNP** | **Alleles** | **Cases**  **(N=1089)** | **% (Cases)** | **Controls (N=1093)** | **% (Controls)** | **OR (95% CI)** | **p-value** |
| --- | --- | --- | --- | --- | --- | --- | --- |
| **rs4253082** | GG | 760 | 69.79 | 814 | 74.54 | 1 | 1 |
|  | **GA** | **308** | **28.28** | **250** | **22.89** | **1.323 (1.089-1.606)** | **0.005** |
|  | AA | 21 | 1.93 | 28 | 2.56 | 0.792 (0.445-1.410) | 0.429 |
| rs958967 | AA | 854 | 78.42 | 829 | 75.99 | 1 | 1 |
|  | AG | 225 | 20.66 | 240 | 22.00 | 0.920 (0.748-1.130) | 0.426 |
|  | GG | 10 | 0.92 | 22 | 2.02 | 0.424 (0.199-0.903) | 0.026 |
| rs4838524 | TT | 854 | 78.42 | 830 | 75.94 | 1 | 1 |
|  | TC | 225 | 20.66 | 241 | 22.05 | 0.916 (0.746-1.126) | 0.405 |
|  | CC | 10 | 0.92 | 22 | 2.01 | 0.424 (0.199-0.904) | 0.026 |
| rs971667 | GG | 687 | 63.09 | 670 | 61.30 | 1 | 1 |
|  | GA | 361 | 33.15 | 360 | 32.94 | 0.978 (0.816-1.173) | 0.813 |
|  | AA | 41 | 3.76 | 63 | 5.76 | 0.640 (0.425-0.963) | 0.032 |
| rs4253126 | CC | 894 | 82.17 | 911 | 83.50 | 1 | 1 |
|  | CA | 190 | 17.46 | 171 | 15.67 | 1.125 (0.897-1.412) | 0.309 |
|  | AA | 4 | 0.37 | 9 | 0.82 | 0.460 (0.141-1.503) | 0.199 |
| rs7076173 | TT | 855 | 78.58 | 833 | 76.35 | 1 | 1 |
|  | TC | 223 | 20.50 | 236 | 21.63 | 0.929 (0.755-1.143) | 0.486 |
|  | CC | 10 | 0.92 | 22 | 2.02 | 0.426 (0.200-0.907) | 0.027 |
| **rs3750749** | TT | 943 | 86.67 | 983 | 89.94 | 1 | 1 |
|  | **TC** | **142** | **13.05** | **107** | **9.79** | **1.396 (1.069-1.823)** | **0.014** |
|  | CC | 3 | 0.28 | 3 | 0.27 | 1.060 (0.213-5.285) | 0.943 |
| rs2229760 | CC | 344 | 31.76 | 361 | 33.15 | 1 | 1 |
|  | CT | 550 | 50.78 | 537 | 49.31 | 1.080 (0.893-1.306) | 0.428 |
|  | TT | 189 | 17.45 | 191 | 17.54 | 1.033 (0.804-1.326) | 0.802 |
| rs2228527 | AA | 685 | 63.13 | 666 | 61.16 | 1 | 1 |
|  | AG | 358 | 33.00 | 359 | 32.97 | 0.970 (0.809-1.163) | 0.741 |
|  | GG | 42 | 3.87 | 64 | 5.88 | 0.644 (0.430-0.966) | 0.033 |
| rs4838519 | CC | 320 | 29.41 | 317 | 29.03 | 1 | 1 |
|  | CA | 550 | 50.55 | 525 | 48.08 | 1.042 (0.856-1.268) | 0.684 |
|  | AA | 218 | 20.04 | 250 | 22.89 | 0.865 (0.680-1.099) | 0.235 |
| rs2228529 | AA | 689 | 63.27 | 669 | 61.32 | 1 | 1 |
|  | AG | 358 | 32.87 | 359 | 32.91 | 0.969 (0.808-1.162) | 0.733 |
|  | GG | 42 | 3.86 | 63 | 5.77 | 0.653 (0.435-0.980) | 0.040 |
| **rs3750751** | GG | 903 | 82.92 | 914 | 83.62 | 1 | 1 |
|  | GA | 171 | 15.70 | 174 | 15.92 | 0.995 (0.790-1.253) | 0.965 |
|  | **AA** | **15** | **1.38** | **5** | **0.46** | **2.911 (1.051-8.064)** | **0.040** |
|  |  |  |  |  |  |  |  |
| ***ERCC8* SNP** | **Alleles** | **Cases**  **(N=1089)** | **% (Cases)** | **Controls (N=1093)** | **% (Controls)** | **OR (95% CI)** | **p-value** |
| rs12520314 | TT | 1006 | 92.89 | 994 | 91.61 | 1 | 1 |
|  | TC | 73 | 6.74 | 89 | 8.20 | 0.828 (0.600-1.1430) | 0.251 |
|  | CC | 4 | 0.37 | 2 | 0.18 | 2.017 (0.367-11.069) | 0.420 |
| rs4647128 | AA | 992 | 92.36 | 1017 | 94.08 | 1 | 1 |
|  | AG | 78 | 7.26 | 64 | 5.92 | 1.267 (0.899-1.785) | 0.176 |
|  | GG | 4 | 0.37 | 0 | 0.00 | >999.999 | 0.971 |

Unconditional logistic regression models adjusted for family history and restricted to Caucasian women ≥ 55 years of age

| ***ERCC6* Haplotype** | **Haplotype number** | **Cases  (N=2178)** | **Case %** | **Controls  (N=2186)** | **Control %** | **OR (95% CI)** | **p-value** |
| --- | --- | --- | --- | --- | --- | --- | --- |
| GACATTTCGTAG | 0 | 933 | 42.84 | 920 | 42.09 | 1 | 1 |
| GGAGCTTCATAG | 1 | 440 | 20.20 | 485 | 22.19 | 0.897 (0.766-1.051) | 0.178 |
| GAAACTCCGCGG | 2 | 193 | 8.86 | 227 | 10.38 | 0.838 (0.677-1.037) | 0.104 |
| GAAACTTAGTAA | 3 | 196 | 9.00 | 191 | 8.74 | 1.010 (0.810-1.258) | 0.932 |
| AACACTTCGTAG | 4 | 200 | 9.18 | 183 | 8.37 | 1.074 (0.861-1.339) | 0.528 |
| **GAAACCTCGTAA** | **5** | **148** | **6.80** | **113** | **5.17** | **1.303 (1.003-1.693)** | **0.048** |
| GACACTCCGCGG | 6 | 49 | 2.25 | 53 | 2.42 | 0.926 (0.621-1.382) | 0.708 |
| Rare* | 7 | 19 | 0.87 | 14 | 0.64 | 1.341 (0.667-2.695) | 0.410 |
|  | | | | | | | |
| Unconditional logistic regression models adjusted for family history and restricted to Caucasian women ≥ 55 years  *Rare haplotypes with individual frequencies of <2% among the controls: | | | | | | | |
| GAAACTTCGTAA |  |  |  |  |  |  |  |
| GAAACTTCGCGG |  |  |  |  |  |  |  |
| GACACTTCGTAG |  |  |  |  |  |  |  |
| GAAGCTTCGCGG |  |  |  |  |  |  |  |
| GGAGCTTCGTAG |  |  |  |  |  |  |  |
| AACATTTCGTAG |  |  |  |  |  |  |  |
| GAAACTTCGTAG |  |  |  |  |  |  |  |
| GACATTTCATAG |  |  |  |  |  |  |  |
| GAAACTTAATAA |  |  |  |  |  |  |  |
| GAAATTTAGTAA |  |  |  |  |  |  |  |
| GGAGCTCCGCGG |  |  |  |  |  |  |  |

| ***ERCC6* Diplotype** | **Diplotype number** | **Cases  (N=1089)** | **Case %** | **Controls  (N=1093)** | **Control %** | **OR (95% CI)** | **p-value** |
| --- | --- | --- | --- | --- | --- | --- | --- |
| GACATTTCGTAG,GACATTTCGTAG | 0,0 | 189 | 17.36 | 191 | 17.47 | 1 | 1 |
| GGAGCTTCATAG,GACATTTCGTAG | 1,0 | 193 | 17.72 | 193 | 17.66 | 1.020 (0.767, 1.355) | 0.893 |
| GAAACTCCGCGG,GACATTTCGTAG | 2,0 | 90 | 8.26 | 95 | 8.69 | 0.973 (0.684, 1.385) | 0.880 |
| GAAACTTAGTAA,GACATTTCGTAG | 3,0 | 96 | 8.82 | 88 | 8.05 | 1.103 (0.775, 1.571) | 0.585 |
| GACATTTCGTAG,AACACTTCGTAG | 0,4 | 92 | 8.45 | 86 | 7.87 | 1.090 (0.763, 1.558) | 0.637 |
| GGAGCTTCATAG,GGAGCTTCATAG | 1,1 | 41 | 3.76 | 63 | 5.76 | 0.668 (0.429, 1.039) | 0.074 |
| GGAGCTTCATAG,GAAACTCCGCGG | 1,2 | 38 | 3.49 | 54 | 4.94 | 0.707 (0.445, 1.123) | 0.142 |
| GGAGCTTCATAG,AACACTTCGTAG | 1,4 | 31 | 2.85 | 43 | 3.93 | 0.731 (0.441, 1.211) | 0.223 |
| GAAACCTCGTAA,GACATTTCGTAG | 5,0 | 57 | 5.23 | 43 | 3.93 | 1.361 (0.872, 2.124) | 0.175 |
| GGAGCTTCATAG,GAAACTTAGTAA | 1,3 | 41 | 3.76 | 35 | 3.20 | 1.193 (0.727, 1.957) | 0.485 |
| GACACTCCGCGG,GACATTTCGTAG | 6,0 | 17 | 1.56 | 29 | 2.65 | 0.608 (0.323, 1.145) | 0.123 |
| GGAGCTTCATAG,GAAACCTCGTAA | 1,5 | 33 | 3.03 | 22 | 2.01 | 1.525 (0.856, 2.717) | 0.152 |
| GAAACTTAGTAA,GAAACTCCGCGG | 3,2 | 14 | 1.29 | 19 | 1.74 | 0.756 (0.368, 1.556) | 0.448 |
| GAAACTCCGCGG,AACACTTCGTAG | 2,4 | 12 | 1.10 | 16 | 1.46 | 0.761 (0.350, 1.655) | 0.491 |
| GAAACTTAGTAA,GAAACCTCGTAA | 3,5 | 13 | 1.19 | 16 | 1.46 | 0.796 (0.371, 1.705) | 0.557 |
| GAAACTCCGCGG,GAAACTCCGCGG | 2,2 | 8 | 0.73 | 15 | 1.37 | 0.522 (0.216, 1.264) | 0.150 |
| GAAACCTCGTAA,AACACTTCGTAG | 5,4 | 14 | 1.29 | 15 | 1.37 | 0.963 (0.451, 2.053) | 0.922 |
| GAAACTTAGTAA,GAAACTTAGTAA | 3,3 | 4 | 0.37 | 9 | 0.82 | 0.460 (0.139, 1.523) | 0.204 |
| GAAACTTAGTAA,AACACTTCGTAG | 3,4 | 17 | 1.56 | 9 | 0.82 | 1.914 (0.831, 4.411) | 0.127 |
| **GAAACTCCGCGG,GAAACCTCGTAA** | **2,5** | **20** | **1.84** | **8** | **0.73** | **2.648 (1.137, 6.170)** | **0.024** |
| **GGAGCTTCATAG,GACACTCCGCGG** | **1,6** | **19** | **1.74** | **5** | **0.46** | **3.893 (1.422,10.658)** | **0.008** |
| GAAACTCCGCGG,GACACTCCGCGG | 2,6 | 2 | 0.18 | 5 | 0.46 | 0.373 (0.071, 1.959) | 0.244 |
| **AACACTTCGTAG,AACACTTCGTAG** | **4,4** | **15** | **1.38** | **5** | **0.46** | **2.922 (1.038, 8.223)** | **0.042** |
| GAAACTTAGTAA,GACACTCCGCGG | 3,6 | 4 | 0.37 | 4 | 0.37 | 1.003 (0.246, 4.086) | 0.997 |
| GAAACCTCGTAA,GAAACCTCGTAA | 5,5 | 3 | 0.28 | 3 | 0.27 | 1.034 (0.205, 5.207) | 0.968 |
| GAAACCTCGTAA,GACACTCCGCGG | 5,6 | 4 | 0.37 | 3 | 0.27 | 1.465 (0.323, 6.639) | 0.621 |
| GACACTCCGCGG,AACACTTCGTAG | 6,4 | 3 | 0.28 | 3 | 0.27 | 1.099 (0.219, 5.516) | 0.909 |
| Rare Diplotypes* |  | 19 | 1.74 | 16 | 1.46 | 1.214 (0.605, 2.437) | 0.585 |
| Unconditional logistic regression models adjusted for family history and restricted to Caucasian women ≥ 55 years of age | | | | | | | |
| *Rare diplotypes with individual frequencies of <2% each among the controls: | | | | | | | |
| GACATTTCGTAG,GAAGCTTCGCGG, |  |  |  |  |  |  |  |
| GACATTTCGTAG,GGAGCTTCGTAG, |  |  |  |  |  |  |  |
| GACATTTCGTAG,GAAACTTAATAA |  |  |  |  |  |  |  |
| GACATTTCGTAG,GAAATTTAGTAA, |  |  |  |  |  |  |  |
| GGAGCTTCATAG,GAAGCTTCGCGG, |  |  |  |  |  |  |  |
| GGAGCTTCATAG,AACATTTCGTAG |  |  |  |  |  |  |  |
| GGAGCTTCATAG,GAAACTTCGTAG, |  |  |  |  |  |  |  |
| GGAGCTTCATAG,GAAACTTCGTAA, |  |  |  |  |  |  |  |
| GGAGCTTCATAG,GAAACTTCGCGG |  |  |  |  |  |  |  |
| GGAGCTTCATAG,GACACTTCGTAG, |  |  |  |  |  |  |  |
| GAAGCTTCGCGG,AACACTTCGTAG, |  |  |  |  |  |  |  |
| GGAGCTTCGTAG,GAAACTTAGTAA |  |  |  |  |  |  |  |
| GGAGCTCCGCGG,GGAGCTTCATAG, |  |  |  |  |  |  |  |
| GAAACTCCGCGG,GAAACTTCGTAA, |  |  |  |  |  |  |  |
| GAAACTTAGTAA,GACATTTCATAG |  |  |  |  |  |  |  |
| GAAACTTAGTAA,GAAACTTAATAA, |  |  |  |  |  |  |  |
| AACACTTCGTAG,GAAACTTCGTAA, |  |  |  |  |  |  |  |
| GACACTCCGCGG,GACACTCCGCGG |  |  |  |  |  |  |  |
| GAAACTTCGTAA,GACATTTCGTAG, |  |  |  |  |  |  |  |
| GAAACTTCGCGG,GACATTTCGTAG, |  |  |  |  |  |  |  |
| GACACTTCGTAG,GACATTTCGTAG |  |  |  |  |  |  |  |
| GACACTTCGTAG,GAAACTTAGTAA, |  |  |  |  |  |  |  |
| GACACTTCGTAG,GAAACCTCGTAA |  |  |  |  |  |  |  |

| ***ERCC8* Haplotype** | **Haplotype number** | **Cases  (N=2178)** | **Case %** | **Controls  (N=2186)** | **Control %** | **OR (95% CI)** | **p-value** |  |
| --- | --- | --- | --- | --- | --- | --- | --- | --- |
| AT | 1 | 2011 | 92.33 | 2029 | 92.82 | 1 | 1 |  |
| AC | 2 | 81 | 3.72 | 93 | 4.25 | 0.897 (0.661-1.216) | 0.483 |  |
| GT | 3 | 86 | 3.95 | 64 | 2.93 | 1.367 (0.983-1.902) | 0.063 |  |
|  | | | | | | | |  |
| Unconditional logistic regression models adjusted for family history and restricted to Caucasian women ≥ 55 years of age | | | | | | | | |

| ***ERCC8* Diplotype** | **Diplotype number** | **Cases  (N=1089)** | | **Case %** | **Controls  (N=1093)** | | **Control %** | **OR (95% CI)** | | **p-value** | |  |  |
| --- | --- | --- | --- | --- | --- | --- | --- | --- | --- | --- | --- | --- | --- |
| AT, AT | 1, 1 | 933 | | 85.67 | 942 | | 86.18 | 1 | | 1 | |  |  |
| AT, AC | 1, 2 | 70 | | 6.43 | 85 | | 7.78 | 0.847 (0.609, 1.178) | | 0.325 | |  |  |
| AT, GT | 1, 3 | 75 | | 6.89 | 60 | | 5.49 | 1.278 (0.898, 1.817) | | 0.173 | |  |  |
| Rare |  | 11 | | 1.01 | 60 | | 0.55 | 1.882 (0.692, 5.121) | | 0.216 | |  |  |
|  | | | | | | | | | | | | |  |
| Unconditional logistic regression models adjusted for family history and restricted to Caucasian women ≥ 55 years of age | | | | | | | | | | | | | |
|  |  |  | |  |  | |  |  | |  | |  |  |
| Rare diplotypes: | | |  |  | |  |  | |  | |  | |  |
| AC,AC |  |  | |  |  | |  |  | |  | |  |  |
| AC,GT |  |  | |  |  | |  |  | |  | |  |  |
| GT,GT |  |  | |  |  | |  |  | |  | |  |  |

**Supplementary** **Table 3. Single Nucleotide Polymorphism (SNP), Haplotype, and Diplotype Analysis in National**

**Surgical Adjuvant Breast and Bowel Project (NSABP)**

| ***ERCC6* SNP** | **Alleles** | **Cases**  **(N=430)** | **% (Cases)** | **Controls (N=822)** | **% (Controls)** | **OR (95% CI)** | **p-value** |
| --- | --- | --- | --- | --- | --- | --- | --- |
| rs3793786 | AA | 241 | 56.05 | 442 | 53.77 | 1 |  |
|  | AG | 167 | 38.84 | 320 | 38.93 | 0.959 (0.749-1.228) | 0.741 |
|  | GG | 22 | 5.12 | 60 | 7.30 | 0.684 (0.406-1.152) | 0.154 |
| rs4253042 | AA | 253 | 58.84 | 510 | 62.04 | 1 |  |
|  | AG | 162 | 37.67 | 274 | 33.33 | 1.176 (0.919-1.506) | 0.197 |
|  | GG | 15 | 3.49 | 38 | 4.62 | 0.781 (0.422-1.445) | 0.431 |
| rs2228528 | GG | 298 | 69.30 | 572 | 69.59 | 1 |  |
|  | GA | 124 | 28.84 | 231 | 28.10 | 1.039 (0.801-1.347) | 0.773 |
|  | AA | 8 | 1.86 | 19 | 2.31 | 0.815 (0.345-1.927) | 0.641 |
| rs4253060 | GG | 380 | 88.37 | 724 | 88.08 | 1 |  |
|  | GA | 49 | 11.40 | 93 | 11.31 | 1.013 (0.693-1.480) | 0.949 |
|  | AA | 1 | 0.23 | 5 | 0.61 | 0.401 (0.047-3.434) | 0.404 |
| rs4253077 | CC | 361 | 83.95 | 660 | 80.29 | 1 |  |
|  | CA | 67 | 15.58 | 155 | 18.86 | 0.802 (0.590-1.090) | 0.159 |
|  | AA | 2 | 0.47 | 7 | 0.85 | 0.539 (0.112-2.600) | 0.441 |
| rs4253082 | GG | 297 | 69.07 | 570 | 69.34 | 1 |  |
|  | GA | 125 | 29.07 | 233 | 28.35 | 1.036 (0.799-1.342) | 0.790 |
|  | AA | 8 | 1.86 | 19 | 2.31 | 0.814 (0.344-1.925) | 0.639 |
| rs958967 | AA | 343 | 79.77 | 623 | 75.79 | 1 |  |
|  | AG | 84 | 19.53 | 189 | 22.99 | 0.812 (0.612-1.078) | 0.151 |
|  | GG | 3 | 0.70 | 10 | 1.22 | 0.569 (0.156-2.071) | 0.392 |
| rs4838524 | AA | 343 | 79.77 | 623 | 75.79 | 1 |  |
|  | AG | 84 | 19.53 | 189 | 22.99 | 0.812 (0.612-1.078) | 0.151 |
|  | GG | 3 | 0.70 | 10 | 1.22 | 0.569 (0.156-2.071) | 0.392 |
| rs971667 | GG | 252 | 58.60 | 510 | 62.04 | 1 |  |
|  | GA | 163 | 37.91 | 273 | 33.21 | 1.193 (0.931-1.527) | 0.162 |
|  | AA | 15 | 3.49 | 39 | 4.74 | 0.766 (0.415-1.414) | 0.395 |
| rs4253126 | CC | 345 | 80.23 | 667 | 81.14 | 1 |  |
|  | CA | 81 | 18.84 | 145 | 17.64 | 1.087 (0.806-1.466) | 0.585 |
|  | AA | 4 | 0.93 | 10 | 1.22 | 0.766 (0.239-2.456) | 0.654 |
| rs7076173 | AA | 344 | 80.00 | 628 | 76.40 | 1 |  |
|  | AG | 83 | 19.30 | 184 | 22.38 | 0.826 (0.621-1.099) | 0.190 |
|  | GG | 3 | 0.70 | 10 | 1.22 | 0.571 (0.157-2.079) | 0.395 |
| rs3750749 | AA | 380 | 88.37 | 723 | 87.96 | 1 |  |
|  | AG | 49 | 11.40 | 94 | 11.44 | 10.685-1.460) | 1.000 |
|  | GG | 1 | 0.23 | 5 | 0.61 | 0.400 (0.047-3.427) | 0.403 |
| **rs2229760** | GG | 137 | 31.86 | 316 | 38.44 | 1 |  |
|  | **GA** | **216** | **50.23** | **363** | **44.16** | **1.403 (1.077-1.827)** | **0.012** |
|  | AA | 77 | 17.91 | 143 | 17.40 | 1.278 (0.904-1.807) | 0.165 |
| rs2228527 | AA | 253 | 58.84 | 509 | 61.92 | 1 |  |
|  | AG | 162 | 37.67 | 274 | 33.33 | 1.174 (0.917-1.503) | 0.204 |
|  | GG | 15 | 3.49 | 39 | 4.74 | 0.761 (0.413-1.405) | 0.383 |
| rs4838519 | CC | 120 | 27.91 | 224 | 27.25 | 1 |  |
|  | CA | 216 | 50.23 | 404 | 49.15 | 1.009 (0.769-1.325) | 0.948 |
|  | AA | 94 | 21.86 | 194 | 23.60 | 0.899 (0.646-1.252) | 0.529 |
| rs2228529 | AA | 253 | 58.84 | 508 | 61.88 | 1 |  |
|  | AG | 162 | 37.67 | 274 | 33.37 | 1.171 (0.914-1.499) | 0.211 |
|  | GG | 15 | 3.49 | 39 | 4.75 | 0.760 (0.412-1.403) | 0.381 |
| rs3750751 | GG | 373 | 86.74 | 672 | 81.75 | 1 |  |
|  | GA | 52 | 12.09 | 146 | 17.76 | 0.627 (0.445-0.884) | 0.008 |
|  | AA | 5 | 1.16 | 4 | 0.49 | 2.526 (0.587-10.872) | 0.213 |

| ***ERCC8* SNP** | **Alleles** | **Cases**  **(N=430)** | **% (Cases)** | **Controls (N=822)** | **% (Controls)** | **OR (95% CI)** | **p-value** |
| --- | --- | --- | --- | --- | --- | --- | --- |
| rs12520314 | AA | 401 | 93.26 | 762 | 92.70 | 1 |  |
|  | AG | 29 | 6.74 | 60 | 7.30 | 0.922 (0.584-1.457) | 0.730 |
| rs158570 | AA | 165 | 38.37 | 300 | 36.50 | 1 |  |
|  | AG | 207 | 48.14 | 406 | 49.39 | 0.931 (0.723-1.198) | 0.577 |
|  | GG | 58 | 13.49 | 116 | 14.11 | 0.918 (0.634-1.330) | 0.652 |
| rs4647128 | AA | 401 | 93.26 | 758 | 92.21 | 1 |  |
|  | AG | 28 | 6.51 | 62 | 7.54 | 0.870 (0.545-1.388) | 0.559 |
|  | GG | 1 | 0.23 | 2 | 0.24 | 0.978 (0.089-10.797) | 0.985 |

| ***ERCC6* Haplotype** | **Haplotype  number** | **Case  (N=860)** | **Case %** | **Control  (N=1644)** | **Control %** | **OR (95% CI)** | **p-value** |
| --- | --- | --- | --- | --- | --- | --- | --- |
| GACAAAACGAAGCGGAA | 0 | 369 | 42.91 | 649 | 39.48 | 1 | 1 |
| GGAGGAACAAAGCGGGA | 1 | 192 | 22.33 | 349 | 21.23 | 0.948 (0.762-1.180) | 0.635 |
| GAAAGAAAGAAACGAAG | 2 | 89 | 10.35 | 165 | 10.04 | 0.939 (0.703-1.253) | 0.667 |
| GAAAGAGCGGGGAGGAG | 3 | 69 | 8.02 | 163 | 9.91 | 0.743 (0.546-1.013) | 0.060 |
| AACAGAACGAAGCGGAA | 4 | 62 | 7.21 | 153 | 9.31 | 0.689 (0.498-0.954) | 0.025 |
| GAAAGGACGAAACAAAG | 5 | 50 | 5.81 | 103 | 6.27 | 0.855 (0.592-1.234) | 0.402 |
| GACAGAGCGGGGCGGAA | 6 | 20 | 2.33 | 40 | 2.43 | 0.856 (0.492-1.490) | 0.583 |
| GACAGAACGAAGCGGAA | 7 | 4 | 0.47 | 10 | 0.61 | 0.698 (0.217-2.242) | 0.546 |
| Rare* | 8 | 5 | 0.58 | 12 | 0.73 | 0.690 (0.238-2.004) | 0.495 |
| Conditional logistic regression models (matching factors: age at trial entry, time in the study, history of lobular carcinoma in situ, and 5-year predicted breast cancer risk based on the Gail model) restricted to Caucasian subjects ≥50 years of age  *Rare haplotypes with individual frequencies of <2% each among the controls: | | | | | | | |
| GAAAGAACGGGGAGGAG |  |  |  |  |  |  |  |
| GAAAGAACGAAACGGAG |  |  |  |  |  |  |  |
| GGAGGAACAAAGCGGAA |  |  |  |  |  |  |  |
| GGAGGAACGAAGCGGGA |  |  |  |  |  |  |  |
| GAAAGGACGAAACGAAG |  |  |  |  |  |  |  |
| AAAAGAGCGGGGAGGAG |  |  |  |  |  |  |  |
| GAAAGAACGAAGAGGAG |  |  |  |  |  |  |  |
| GAAAGGACGAAACAAAA |  |  |  |  |  |  |  |

| ***ERCC6* Diplotype** | **Diplotype number** | **Case  (N=430)** | **Case %** | **Control  (N=822)** | **Control %** | **OR (95% CI)** | **p-value** |
| --- | --- | --- | --- | --- | --- | --- | --- |
| GACAAAACGAAGCGGAA,GACAAAACGAAGCGGAA | 0,0 | 77 | 17.91 | 143 | 17.40 | 1 | 1 |
| GGAGGAACAAAGCGGGA,GACAAAACGAAGCGGAA | 1,0 | 87 | 20.23 | 129 | 15.69 | 1.234 (0.837-1.819) | 0.288 |
| GAAAGAGCGGGGAGGAG,GACAAAACGAAGCGGAA | 3,0 | 30 | 6.98 | 65 | 7.91 | 0.846 (0.506-1.415) | 0.525 |
| GAAAGAAAGAAACGAAG,GACAAAACGAAGCGGAA | 2,0 | 38 | 8.84 | 64 | 7.79 | 1.082 (0.671-1.744) | 0.746 |
| GACAAAACGAAGCGGAA,AACAGAACGAAGCGGAA | 0,4 | 26 | 6.05 | 56 | 6.81 | 0.824 (0.476-1.426) | 0.488 |
| GGAGGAACAAAGCGGGA,GAAAGAAAGAAACGAAG | 1,2 | 25 | 5.81 | 39 | 4.74 | 1.175 (0.661-2.086) | 0.583 |
| GGAGGAACAAAGCGGGA,GGAGGAACAAAGCGGGA | 1,1 | 15 | 3.49 | 38 | 4.62 | 0.697 (0.358-1.358) | 0.289 |
| GAAAGGACGAAACAAAG,GACAAAACGAAGCGGAA | 5,0 | 22 | 5.12 | 33 | 4.01 | 1.278 (0.689-2.369) | 0.437 |
| GGAGGAACAAAGCGGGA,AACAGAACGAAGCGGAA | 1,4 | 9 | 2.09 | 32 | 3.89 | 0.517 (0.235-1.137) | 0.101 |
| GGAGGAACAAAGCGGGA,GAAAGAGCGGGGAGGAG | 1,3 | 19 | 4.42 | 31 | 3.77 | 1.051 (0.553-2.000) | 0.879 |
| GGAGGAACAAAGCGGGA,GAAAGGACGAAACAAAG | 1,5 | 12 | 2.79 | 21 | 2.55 | 1.081 (0.479-2.440) | 0.851 |
| GAAAGAAAGAAACGAAG,AACAGAACGAAGCGGAA | 2,4 | 5 | 1.16 | 18 | 2.19 | 0.405 (0.129-1.274) | 0.122 |
| GAAAGAAAGAAACGAAG,GAAAGAGCGGGGAGGAG | 2,3 | 7 | 1.63 | 18 | 2.19 | 0.671 (0.262-1.716) | 0.405 |
| GAAAGAGCGGGGAGGAG,AACAGAACGAAGCGGAA | 3,4 | 4 | 0.93 | 17 | 2.07 | 0.443 (0.143-1.373) | 0.158 |
| GGAGGAACAAAGCGGGA,GACAGAGCGGGGCGGAA | 1,6 | 8 | 1.86 | 16 | 1.95 | 0.873 (0.351-2.169) | 0.770 |
| GAAAGGACGAAACAAAG,AACAGAACGAAGCGGAA | 5,4 | 6 | 1.40 | 14 | 1.70 | 0.776 (0.286-2.103) | 0.618 |
| GAAAGGACGAAACAAAG,GAAAGAGCGGGGAGGAG | 5,3 | 4 | 0.93 | 13 | 1.58 | 0.560 (0.178-1.763) | 0.322 |
| GACAGAGCGGGGCGGAA,GACAAAACGAAGCGGAA | 6,0 | 7 | 1.63 | 10 | 1.22 | 1.078 (0.391-2.973) | 0.885 |
| Rare* |  | 29 | 6.74 | 65 | 7.91 | 0.794 (0.468-1.346) | 0.391 |
| Conditional logistic regression models (matching factors: age at trial entry, time in the study, history of lobular carcinoma in situ, and 5-year predicted breast cancer risk based on the Gail model) restricted to Caucasian subjects ≥50 years of age  *Rare diplotypes with individual frequencies of <2% among the controls: | | | | | | | |
| GGAGGAACAAAGCGGGA,GGAGGAACAAAGCGGAA |  |  |  |  |  |  |  |
| GGAGGAACAAAGCGGGA,AAAAGAGCGGGGAGGAG |  |  |  |  |  |  |  |
| GGAGGAACAAAGCGGGA,GAAAGGACGAAACAAAA |  |  |  |  |  |  |  |
| GGAGGAACAAAGCGGGA,GACAGAACGAAGCGGAA |  |  |  |  |  |  |  |
| GGAGGAACAAAGCGGGA,GAAAGAACGAAACGGAG |  |  |  |  |  |  |  |
| GGAGGAACAAAGCGGAA,GAAAGGACGAAACAAAG |  |  |  |  |  |  |  |
| GGAGGAACGAAGCGGGA,GAAAGGACGAAACAAAG |  |  |  |  |  |  |  |
| GAAAGGACGAAACGAAG,GAAAGAACGGGGAGGAG |  |  |  |  |  |  |  |
| GAAAGAACGAAGAGGAG,GACAAAACGAAGCGGAA |  |  |  |  |  |  |  |
| GAAAGAAAGAAACGAAG,GACAAAACAAAGCGGAA |  |  |  |  |  |  |  |
| GAAAGAAAGAAACGAAG,GAAAGAAAGAAACGAAG |  |  |  |  |  |  |  |
| GAAAGAAAGAAACGAAG,GACAGAGCGGGGCGGAA |  |  |  |  |  |  |  |
| GAAAGAGCGGGGAGGAG,GAAAGAGCGGGGAGGAG |  |  |  |  |  |  |  |
| GAAAGAGCGGGGAGGAG,GACAGAGCGGGGCGGAA |  |  |  |  |  |  |  |
| GAAAGAGCGGGGAGGAG,GACAGAACGAAGCGGAA |  |  |  |  |  |  |  |
| AACAGAACGAAGCGGAA,AACAGAACGAAGCGGAA |  |  |  |  |  |  |  |
| GAAAGGACGAAACAAAG,GAAAGAAAGAAACGAAG |  |  |  |  |  |  |  |
| GAAAGGACGAAACAAAG,GAAAGGACGAAACAAAG |  |  |  |  |  |  |  |
| GAAAGGACGAAACAAAG,GACAGAGCGGGGCGGAA |  |  |  |  |  |  |  |
| GACAGAGCGGGGCGGAA,AACAGAACGAAGCGGAA |  |  |  |  |  |  |  |
| GACAGAACGAAGCGGAA,GACAAAACGAAGCGGAA |  |  |  |  |  |  |  |
| GACAGAACGAAGCGGAA,AACAGAACGAAGCGGAA |  |  |  |  |  |  |  |
| GAAAGAACGGGGAGGAG,GACAAAACGAAGCGGAA |  |  |  |  |  |  |  |
| GAAAGAACGGGGAGGAG,GAAAGAAAGAAACGAAG |  |  |  |  |  |  |  |
| GAAAGAACGGGGAGGAG,AACAGAACGAAGCGGAA |  |  |  |  |  |  |  |
| GAAAGAACGAAACGGAG,GACAAAACGAAGCGGAA |  |  |  |  |  |  |  |
| GAAAGAACGAAACGGAG,AACAGAACGAAGCGGAA |  |  |  |  |  |  |  |

| ***ERCC8* Haplotype** | **Haplotype number** | **Case  (N=860)** | **Case %** | **Control  (N=1644)** | **Control %** | **OR (95% CI)** | **p-value** |
| --- | --- | --- | --- | --- | --- | --- | --- |
| AAA | 0 | 508 | 59.07 | 946 | 57.54 | 1 | 1 |
| AGA | 1 | 293 | 34.07 | 572 | 34.79 | 0.956 (0.801-1.141) | 0.619 |
| GGA | 2 | 30 | 3.49 | 66 | 4.01 | 0.865 (0.552-1.355) | 0.526 |
| AAG | 3 | 29 | 3.37 | 60 | 3.65 | 0.904 (0.574-1.426) | 0.665 |

Conditional logistic regression models (matching factors: age at trial entry, time in the study, history of lobular carcinoma in situ,

and 5-year predicted breast cancer risk based on the Gail model) restricted to Caucasian subjects ≥50 years of age

| ***ERCC8* Diplotype** | **Diplotype  number** | **Case  (N=430)** | **Case %** | **Control  (N=822)** | **Control %** | **OR (95% CI)** | **p-value** |
| --- | --- | --- | --- | --- | --- | --- | --- |
| AGA,AAA | 0,0 | 141 | 32.79 | 269 | 32.73 | 1 | 1 |
| AAA,AAA | 1,0 | 181 | 42.09 | 340 | 41.36 | 0.998 (0.758-1.314) | 0.988 |
| AGA,AGA | 1,1 | 51 | 11.86 | 91 | 11.07 | 1.058 (0.708-1.581) | 0.782 |
| GGA,AAA | 2,0 | 21 | 4.88 | 37 | 4.50 | 1.087 (0.615-1.919) | 0.774 |
| AAA,AAG | 0,3 | 24 | 5.58 | 31 | 3.77 | 1.461 (0.823-2.595) | 0.196 |
| AGA,AAG | 1,3 | 4 | 0.93 | 27 | 3.28 | 0.291 (0.100-0.847) | 0.024 |
| GGA,AGA | 2,1 | 6 | 1.40 | 23 | 2.80 | 0.509 (0.199-1.303) | 0.159 |
| GGA,AAG | 2,3 | 1 | 0.23 | 2 | 0.24 | 1.013 (0.091-11.292) | 0.992 |
| GGA,GGA | 2,2 | 1 | 0.23 | 2 | 0.24 | 0.722 (0.061-8.599) | 0.797 |

Conditional logistic regression models (matching factors: age at trial entry, time in the study, history of lobular carcinoma in situ,

and 5-year predicted breast cancer risk based on the Gail model) restricted to Caucasian subjects ≥50 years of age

**Supplementary** **Table 4. Single Nucleotide Polymorphism (SNP), Haplotype, and Diplotype Analysis in Women's Health**

**Initiative (WHI) Hormone Therapy Trials**

| ***ERCC6* SNP** |  | **Alleles** | **Cases**  **(N=465)** | **% (Cases)** | **Controls (N=1394)** | **% (Controls)** | **Odds Ratio (95% CI)** | **p-value** |
| --- | --- | --- | --- | --- | --- | --- | --- | --- |
| **rs1012553** | 0 | TT | 220 | 51.64 | 752 | 58.57 | 1 |  |
|  | **1** | **TA** | **182** | **42.72** | **455** | **35.44** | **1.351 (1.070-1.706)** | **0.011** |
|  | 2 | AA | 24 | 5.63 | 77 | 6.00 | 1.100 (0.672-1.799) | 0.705 |
| **rs2228528** | 0 | GG | 292 | 68.55 | 940 | 73.10 | 1 |  |
|  | **1** | **GA** | **125** | **29.34** | **316** | **24.57** | **1.292 (1.006-1.660)** | **0.045** |
|  | 2 | AA | 9 | 2.11 | 30 | 2.33 | 1.021 (0.471-2.214) | 0.957 |
| rs1018603 | 0 | AA | 327 | 76.94 | 1020 | 79.44 | 1 |  |
|  | 1 | AC | 91 | 21.41 | 249 | 19.39 | 1.129 (0.857-1.487) | 0.388 |
|  | 2 | CC | 7 | 1.65 | 15 | 1.17 | 1.324 (0.521-3.364) | 0.555 |
| rs4253060 | 0 | GG | 381 | 89.65 | 1148 | 89.34 | 1 |  |
|  | 1 | GA | 43 | 10.12 | 133 | 10.35 | 0.968 (0.668-1.402) | 0.863 |
|  | 2 | AA | 1 | 0.24 | 4 | 0.31 | 0.975 (0.106-8.971) | 0.982 |
| rs4253082 | 0 | GG | 293 | 68.78 | 937 | 72.86 | 1 |  |
|  | 1 | GA | 124 | 29.11 | 318 | 24.73 | 1.267 (0.986-1.628) | 0.064 |
|  | 2 | AA | 9 | 2.11 | 31 | 2.41 | 0.978 (0.453-2.113) | 0.954 |
| rs4838524 | 0 | AA | 328 | 77.00 | 1018 | 79.28 | 1 |  |
|  | 1 | AG | 91 | 21.36 | 251 | 19.55 | 1.114 (0.846-1.466) | 0.443 |
|  | 2 | GG | 7 | 1.64 | 15 | 1.17 | 1.319 (0.519-3.352) | 0.560 |
| rs10776576 | 0 | GG | 293 | 68.78 | 940 | 73.10 | 1 |  |
|  | 1 | GA | 124 | 29.11 | 316 | 24.57 | 1.275 (0.992-1.638) | 0.058 |
|  | 2 | AA | 9 | 2.11 | 30 | 2.33 | 1.018 (0.469-2.206) | 0.965 |
| rs4253132 | 0 | AA | 327 | 76.94 | 1017 | 79.33 | 1 |  |
|  | 1 | AG | 91 | 21.41 | 250 | 19.50 | 1.121 (0.852-1.477) | 0.415 |
|  | 2 | GG | 7 | 1.65 | 15 | 1.17 | 1.320 (0.519-3.356) | 0.560 |
| rs2228529 | 0 | AA | 251 | 59.20 | 751 | 58.49 | 1 |  |
|  | 1 | AG | 157 | 37.03 | 452 | 35.20 | 1.042 (0.822-1.319) | 0.736 |
|  | 2 | GG | 16 | 3.77 | 81 | 6.31 | 0.549 (0.312-0.964) | 0.037 |
| rs4253231 | 0 | AA | 349 | 82.12 | 1071 | 83.48 | 1 |  |
|  | 1 | AG | 71 | 16.71 | 204 | 15.90 | 1.103 (0.816-1.492) | 0.524 |
|  | 2 | GG | 5 | 1.18 | 8 | 0.62 | 1.668 (0.525-5.300) | 0.385 |
|  |  |  |  |  |  |  |  |  |
| ***ERCC8* SNP** |  | **Alleles** | **Cases**  **(N=465)** | **% (Cases)** | **Controls (N=1394)** | **% (Controls)** | **OR (95% CI)** | **p-value** |
| rs12520314 | 0 | AA | 390 | 92.64 | 1175 | 92.01 | 1 |  |
|  | 1 | AG | 31 | 7.36 | 99 | 7.75 | 0.894 (0.582, 1.371) | 0.606 |
|  | 2 | GG | 0 | 0.00 | 3 | 0.24 | <0.001 | 0.981 |
| rs158933 | 0 | GG | 159 | 37.32 | 503 | 39.11 | 1 |  |
|  | 1 | GA | 203 | 47.65 | 596 | 46.35 | 1.101 (0.863, 1.405) | 0.440 |
|  | 2 | AA | 64 | 15.02 | 187 | 14.54 | 1.125 (0.798, 1.586) | 0.501 |
| rs17332991 | 0 | CC | 333 | 78.72 | 1027 | 80.11 | 1 |  |
|  | 1 | CA | 83 | 19.62 | 236 | 18.41 | 1.156 (0.869, 1.537) | 0.320 |
|  | 2 | AA | 7 | 1.66 | 19 | 1.48 | 1.185 (0.483, 2.910) | 0.711 |
| rs3117 | 0 | AA | 160 | 37.74 | 497 | 38.86 | 1 |  |
|  | 1 | AG | 201 | 47.41 | 597 | 46.68 | 1.067 (0.836, 1.363) | 0.600 |
|  | 2 | GG | 63 | 14.86 | 185 | 14.46 | 1.100 (0.779, 1.554) | 0.587 |
| rs4647028 | 0 | CC | 231 | 54.35 | 744 | 57.85 | 1 |  |
|  | 1 | CA | 167 | 39.29 | 460 | 35.77 | 1.174 (0.928, 1.486) | 0.181 |
|  | 2 | AA | 27 | 6.35 | 82 | 6.38 | 1.069 (0.667, 1.712) | 0.783 |
| rs4647128 | 0 | AA | 389 | 92.40 | 1176 | 92.24 | 1 |  |
|  | 1 | AG | 32 | 7.60 | 98 | 7.69 | 1.032 (0.674, 1.579) | 0.885 |
|  | 2 | GG | 0 | 0.00 | 1 | 0.08 | <0.001 | 0.984 |
| rs4647167 | 0 | AA | 231 | 56.89 | 743 | 54.23 | 1 |  |
|  | 1 | AG | 168 | 36.68 | 460 | 39.44 | 1.179 (0.932, 1.492) | 0.169 |
|  | 2 | GG | 27 | 6.43 | 83 | 6.34 | 1.061 (0.662, 1.699) | 0.806 |
| rs976080 | 0 | CC | 358 | 84.04 | 1055 | 82.10 | 1 |  |
|  | 1 | CA | 62 | 14.55 | 216 | 16.81 | 0.849 (0.620, 1.162) | 0.307 |
|  | 2 | AA | 6 | 1.41 | 14 | 1.09 | 1.539 (0.575, 4.118) | 0.391 |

Unconditional logistic regression models adjusting for GRAVID (number of pregnancies), BC_Fam (Breast Cancer Family History),

BMIX (BMI), and BRSTFDMO (Month of Breast Feeding) and restricted to Caucasian subjects ≥50 years of age

| ***ERCC6* Haplotype** | **Haplotype  number** | **Cases  (N=930)** | **Case %** | **Controls  (N=2788)** | **Control %** | **OR (95% CI)** | **p-value** |  |  |  |  |
| --- | --- | --- | --- | --- | --- | --- | --- | --- | --- | --- | --- |
| AAAAAAAGAGGAGG | 0 | 365 | 39.25 | 1149 | 41.21 | 1 | 1 |  |  |  |  |
| AGGGAGAGAGGAGG | 1 | 162 | 17.42 | 516 | 18.51 | 0.984 (0.785, 1.234) | 0.890 |  |  |  |  |
| **AAAAAGAAAAGAAA** | **2** | **101** | **10.86** | **259** | **9.29** | **1.358 (1.034, 1.783)** | **0.028** |  |  |  |  |
| AAAAAGGGGGGCGA | 3 | 92 | 9.89 | 253 | 9.07 | 1.178 (0.889, 1.562) | 0.255 |  |  |  |  |
| GAAAAGAGAGGAGG | 4 | 89 | 9.57 | 237 | 8.50 | 1.204 (0.902, 1.607) | 0.209 |  |  |  |  |
| AAAAAGAAAAAAAA | 5 | 46 | 4.95 | 152 | 5.45 | 1.011 (0.700, 1.460) | 0.954 |  |  |  |  |
| AGGGGGAGAGGAGG | 6 | 48 | 5.16 | 150 | 5.38 | 1.027 (0.713, 1.479) | 0.886 |  |  |  |  |
| AAAAAGGGGGGCGG | 7 | 23 | 2.47 | 58 | 2.08 | 1.153 (0.657, 2.023) | 0.619 |  |  |  |  |
| AAAAAGAGAGGAGG | 8 | 1 | 0.11 | 6 | 0.22 | 0.601 (0.068, 5.290) | 0.646 |  |  |  |  |
| Rare* | 9 | 3 | 0.32 | 8 | 0.29 | 1.487 (0.389, 5.693) | 0.562 |  |  |  |  |
| Unconditional logistic regression models adjusting for GRAVID (number of pregnancies), BC_Fam (Breast Cancer Family History),  BMIX (BMI), and BRSTFDMO (Month of Breast Feeding) and restricted to Caucasian subjects ≥50 years of age  Rare haplotypes with individual frequencies of <2% among the controls: | | | | | | | | | | | |
| AAAAAGAGAAGAGA |  |  |  |  |  |  |  |  |  |  |  |
| AAAAGGAGAGGAGG |  |  |  |  |  |  |  |  |  |  |  |
| AAGGGGAGAGGAGG |  |  |  |  |  |  |  |  |  |  |  |
| AAAAGAAGAGGAGG |  |  |  |  |  |  |  |  |  |  |  |
| AAAAAGAAAGAAAA |  |  |  |  |  |  |  |  |  |  |  |
| AAAAAGAGAAGAAA |  |  |  |  |  |  |  |  |  |  |  |
| AAGAAGGGGGGCGA |  |  |  |  |  |  |  |  |  |  |  |

| ***ERCC6* Diplotype** | **Diplotype number** | **Cases (N=465)** | **Cases %** | **Controls (N=1394)** | **Controls %** | **OR (95% CI)** | **p-value** |
| --- | --- | --- | --- | --- | --- | --- | --- |
| AAAAAAAGAGGAGG,AAAAAAAGAGGAGG | 0,0 | 74 | 15.91 | 236 | 16.93 | 1 | 1 |
| AGGGAGAGAGGAGG,AAAAAAAGAGGAGG | 1,0 | 67 | 14.41 | 207 | 14.85 | 0.954 (0.633, 1.438) | 0.822 |
| AAAAAAAGAGGAGG,AAAAAGAAAAGAAA | 0,2 | 41 | 8.82 | 112 | 8.03 | 1.210 (0.757, 1.935) | 0.426 |
| AAAAAAAGAGGAGG,AAAAAGGGGGGCGA | 0,3 | 36 | 7.74 | 107 | 7.68 | 1.157 (0.712, 1.879) | 0.557 |
| AAAAAAAGAGGAGG,GAAAAGAGAGGAGG | 0,4 | 25 | 5.38 | 96 | 6.89 | 0.876 (0.511, 1.500) | 0.629 |
| AAAAAAAGAGGAGG,AAAAAGAAAAAAAA | 0,5 | 19 | 4.09 | 70 | 5.02 | 0.833 (0.450, 1.541) | 0.561 |
| AGGGGGAGAGGAGG,AAAAAAAGAGGAGG | 6,0 | 18 | 3.87 | 56 | 4.02 | 0.972 (0.516, 1.833) | 0.931 |
| AGGGAGAGAGGAGG,AGGGAGAGAGGAGG | 1,1 | 10 | 2.15 | 55 | 3.95 | 0.586 (0.280, 1.228) | 0.157 |
| AGGGAGAGAGGAGG,GAAAAGAGAGGAGG | 1,4 | 16 | 3.44 | 53 | 3.80 | 1.013 (0.527, 1.947) | 0.968 |
| AGGGAGAGAGGAGG,AAAAAGGGGGGCGA | 1,3 | 19 | 4.09 | 46 | 3.30 | 1.330 (0.706, 2.503) | 0.377 |
| AGGGAGAGAGGAGG,AAAAAGAAAAGAAA | 1,2 | 17 | 3.66 | 41 | 2.94 | 1.611 (0.834, 3.115) | 0.156 |
| AGGGAGAGAGGAGG,AGGGGGAGAGGAGG | 1,6 | 7 | 1.51 | 27 | 1.94 | 0.733 (0.283, 1.897) | 0.522 |
| AAAAAAAGAGGAGG,AAAAAGGGGGGCGG | 0,7 | 9 | 1.94 | 26 | 1.87 | 0.797 (0.309, 2.056) | 0.639 |
| AAAAAGAAAAGAAA,AAAAAGGGGGGCGA | 2,3 | 10 | 2.15 | 26 | 1.87 | 1.185 (0.522, 2.687) | 0.685 |
| AGGGAGAGAGGAGG,AAAAAGAAAAAAAA | 1,5 | 12 | 2.58 | 24 | 1.72 | 1.718 (0.798, 3.697) | 0.167 |
| **AAAAAGAAAAGAAA,GAAAAGAGAGGAGG** | **2,4** | **12** | **2.58** | **18** | **1.29** | **2.482 (1.111, 5.547)** | **0.027** |
| AAAAAGGGGGGCGA,GAAAAGAGAGGAGG | 3,4 | 12 | 2.58 | 17 | 1.22 | 1.889 (0.811, 4.398) | 0.140 |
| Rare* |  | 61 | 13.12 | 177 | 12.70 | 1.169 (0.772, 1.772) | 0.461 |
| Unconditional logistic regression models adjusting for GRAVID (number of pregnancies), BC_Fam (Breast Cancer Family History), BMIX (BMI), and BRSTFDMO (Month of Breast Feeding) and restricted to Caucasian subjects ≥50 years of age  Rare diplotypes with individual frequencies of <2% among the controls: | | | | | | | |
| AAAAAAAGAGGAGG,AAAAAGAGAAGAAA |  |  |  |  |  |  |  |
| AAAAAGAAAGAAAA,GAAAAGAGAGGAGG |  |  |  |  |  |  |  |
| AAGAAGGGGGGCGA,AGGGAGAGAGGAGG |  |  |  |  |  |  |  |
| AAAAAGAAAAGAAA,AAAAAGAAAAGAAA |  |  |  |  |  |  |  |
| AGGGGGAGAGGAGG,AAAAAGGGGGGCGA |  |  |  |  |  |  |  |
| AAAAAAAGAGGAGG,AAAAAGAGAGGAGG |  |  |  |  |  |  |  |
| AAAAAGAGAAGAGA,AAAAGAAGAGGAGG |  |  |  |  |  |  |  |
| AAAAAGAGAAGAGA,AAAAAGGGGGGCGA |  |  |  |  |  |  |  |
| AAAAAGAGAAGAGA,GAAAAGAGAGGAGG |  |  |  |  |  |  |  |
| AAAAAGAGAAGAGA,AAAAAGAAAAAAAA |  |  |  |  |  |  |  |
| AAAAGGAGAGGAGG,GAAAAGAGAGGAGG |  |  |  |  |  |  |  |
| AAGGGGAGAGGAGG,AAAAAAAGAGGAGG |  |  |  |  |  |  |  |
| AGGGAGAGAGGAGG,AAAAAGGGGGGCGG |  |  |  |  |  |  |  |
| AAAAAGAAAAGAAA,AAAAAGAAAAAAAA |  |  |  |  |  |  |  |
| AAAAAGAAAAGAAA,AAAAAGGGGGGCGG |  |  |  |  |  |  |  |
| AAAAAGGGGGGCGA,AAAAGGAGAGGAGG |  |  |  |  |  |  |  |
| AAAAAGGGGGGCGA,AAAAAGGGGGGCGA |  |  |  |  |  |  |  |
| GAAAAGAGAGGAGG,GAAAAGAGAGGAGG |  |  |  |  |  |  |  |
| AAAAAGAAAAAAAA,AAAAAGGGGGGCGA |  |  |  |  |  |  |  |
| AAAAAGAAAAAAAA,GAAAAGAGAGGAGG |  |  |  |  |  |  |  |
| AAAAAGAAAAAAAA,AAAAAGAAAAAAAA |  |  |  |  |  |  |  |
| AAAAAGAAAAAAAA,AAAAAGGGGGGCGG |  |  |  |  |  |  |  |
| AGGGGGAGAGGAGG,AAAAAGAAAAGAAA |  |  |  |  |  |  |  |
| AGGGGGAGAGGAGG,GAAAAGAGAGGAGG |  |  |  |  |  |  |  |
| AGGGGGAGAGGAGG,AAAAAGAAAAAAAA |  |  |  |  |  |  |  |
| AGGGGGAGAGGAGG,AGGGGGAGAGGAGG |  |  |  |  |  |  |  |
| AGGGGGAGAGGAGG,AAAAAGGGGGGCGG |  |  |  |  |  |  |  |
| AAAAAGGGGGGCGG,AAAAAGGGGGGCGA |  |  |  |  |  |  |  |
| AAAAAGGGGGGCGG,GAAAAGAGAGGAGG |  |  |  |  |  |  |  |
| AAAAAGGGGGGCGG,AAAAAGGGGGGCGG |  |  |  |  |  |  |  |
| AAAAAGAGAGGAGG,AAAAAGAAAAGAAA |  |  |  |  |  |  |  |
| AAAAAGAGAGGAGG,GAAAAGAGAGGAGG |  |  |  |  |  |  |  |
| AAAAAGAGAGGAGG,AAAAAGAAAAAAAA |  |  |  |  |  |  |  |

| ***ERCC8* Haplotype** | **Haplotype  number** | **Cases (N=930)** | **Cases %** | **Controls (N=2788)** | **Controls %** | **OR (95% CI)** | **p-value** |  |  |
| --- | --- | --- | --- | --- | --- | --- | --- | --- | --- |
| AACACAGC | 0 | 533 | 57.31 | 1625 | 58.29 | 1 | 1 |  |  |
| GGCACAAA | 1 | 137 | 14.73 | 383 | 13.74 | 1.061 (0.841, 1.340) | 0.618 |  |  |
| GGAACAAA | 2 | 105 | 11.29 | 295 | 10.58 | 1.145 (0.884, 1.482) | 0.305 |  |  |
| AGCAAAAC | 3 | 81 | 8.71 | 261 | 9.36 | 0.951 (0.714, 1.267) | 0.731 |  |  |
| AACACGGC | 4 | 34 | 3.66 | 111 | 3.98 | 0.872 (0.572, 1.328) | 0.523 |  |  |
| AGCGCAAC | 5 | 35 | 3.76 | 106 | 3.80 | 1.028 (0.675, 1.565) | 0.899 |  |  |
| Rare* | 6 | 5 | 0.54 | 7 | 0.25 | 2.568 (0.756, 8.724) | 0.131 |  |  |
| Unconditional logistic regression models adjusting for GRAVID (number of pregnancies), BC_Fam (Breast Cancer Family History),  BMIX (BMI), and BRSTFDMO (Month of Breast Feeding) and restricted to Caucasian subjects ≥50 years of age  * Rare haplotypes with individual frequencies of <2% among the controls: | | | | | | | | | |
| AACACAAC |  |  |  |  |  |  |  |  |  |
| AGCAAAGC |  |  |  |  |  |  |  |  |  |
| GGCACAGC |  |  |  |  |  |  |  |  |  |
| GGCAAAAC |  |  |  |  |  |  |  |  |  |
| GGAACAGC |  |  |  |  |  |  |  |  |  |
| AAAACAGC |  |  |  |  |  |  |  |  |  |
| AACAAAAC |  |  |  |  |  |  |  |  |  |

| ***ERCC8* Diplotype** | **Diplotype number** | **Cases (N=465)** | **Cases %** | **Controls (N=1394)** | **Controls %** | **OR (95% CI)** | **p-value** |  | |  |
| --- | --- | --- | --- | --- | --- | --- | --- | --- | --- | --- |
| AACACAGC,AACACAGC | 0,0 | 146 | 31.40 | 480 | 34.43 | 1 | 1 |  | |  |
| AACACAGC,GGCACAAA | 0,1 | 82 | 17.63 | 211 | 15.14 | 1.226 (0.873, 1.720) | 0.239 |  | |  |
| AACACAGC,GGAACAAA | 0,2 | 67 | 14.41 | 171 | 12.27 | 1.364 (0.954, 1.949) | 0.089 |  | |  |
| AACACAGC,AGCAAAAC | 0,3 | 44 | 9.46 | 161 | 11.55 | 0.850 (0.564, 1.282) | 0.439 |  | |  |
| AACACAGC,AACACGGC | 0,4 | 24 | 5.16 | 61 | 4.38 | 1.169 (0.687, 1.991) | 0.564 |  | |  |
| AACACAGC,AGCGCAAC | 0,5 | 20 | 4.30 | 59 | 4.23 | 1.043 (0.578, 1.882) | 0.889 |  | |  |
| GGCACAAA,GGAACAAA | 1,2 | 11 | 2.37 | 38 | 2.73 | 1.041 (0.496, 2.184) | 0.915 |  | |  |
| AGCAAAAC,GGCACAAA | 3,1 | 10 | 2.15 | 34 | 2.44 | 0.847 (0.390, 1.841) | 0.676 |  | |  |
| GGCACAAA,GGCACAAA | 1,1 | 10 | 2.15 | 30 | 2.15 | 1.041 (0.479, 2.261) | 0.919 |  | |  |
| AGCAAAAC,GGAACAAA | 3,2 | 8 | 1.72 | 23 | 1.65 | 1.036 (0.403, 2.664) | 0.942 |  | |  |
| GGAACAAA,GGAACAAA | 2,2 | 7 | 1.51 | 21 | 1.51 | 1.254 (0.505, 3.114) | 0.626 |  | |  |
| AACACGGC,GGCACAAA | 4,1 | 8 | 1.72 | 20 | 1.43 | 1.270 (0.512, 3.150) | 0.605 |  | |  |
| AGCGCAAC,GGCACAAA | 5,1 | 6 | 1.29 | 17 | 1.22 | 1.178 (0.441, 3.150) | 0.744 |  | |  |
| AGCAAAAC,AGCAAAAC | 3,3 | 6 | 1.29 | 16 | 1.15 | 1.713 (0.632, 4.640) | 0.290 |  | |  |
| AACACGGC,GGAACAAA | 4,2 | 1 | 0.22 | 12 | 0.86 | <0.001 | 0.983 |  | |  |
| AACACGGC,AGCGCAAC | 4,5 | 0 | 0.00 | 10 | 0.72 | <0.001 | 0.986 |  | |  |
| AGCAAAAC,AGCGCAAC | 3,5 | 6 | 1.29 | 10 | 0.72 | 2.290 (0.777 ,6.749) | 0.133 |  | |  |
| AGCGCAAC,GGAACAAA | 5,2 | 3 | 0.65 | 8 | 0.57 | 1.346 (0.341 ,5.316) | 0.672 |  | |  |
| AACACAGC,AACACAAC | 0,6 | 2 | 0.43 | 1 | 0.07 | 6.816 (0.579 ,80.222) | 0.127 |  | |  |
| AACACGGC,AGCAAAAC | 4,3 | 1 | 0.22 | 1 | 0.07 | 3.062 (0.188 ,49.832) | 0.432 |  | |  |
| Rare |  | 3 | 0.65 | 10 | 0.72 | 1.193 (0.309 ,4.601) | 0.798 |  | |  |
|  | | | | | | | | |  | |
| Unconditional logistic regression models adjusting for GRAVID (number of pregnancies), BC_Fam (Breast Cancer Family History),  BMIX (BMI), and BRSTFDMO (Month of Breast Feeding) and restricted to Caucasian subjects ≥50 years of age  Rare diplotypes with individual frequencies of <2% among the controls: | | | | | | | | | | |
| AACACAGC,GGAACAGC, |  |  |  |  |  |  |  |  | |  |
| AACACAGC,AAAACAGC |  |  |  |  |  |  |  |  | |  |
| AACAAAAC,AACACAGC |  |  |  |  |  |  |  |  | |  |
| GGCACAAA,GGCAAAAC |  |  |  |  |  |  |  |  | |  |
| AACACGGC,AACACGGC |  |  |  |  |  |  |  |  | |  |
| AACACGGC,GGCACAGC |  |  |  |  |  |  |  |  | |  |
| AGCGCAAC,AGCGCAAC |  |  |  |  |  |  |  |  | |  |
| AACACAAC,GGCACAAA |  |  |  |  |  |  |  |  | |  |
| AACACAAC,GGAACAAA |  |  |  |  |  |  |  |  | |  |
| AGCAAAGC,GGAACAAA |  |  |  |  |  |  |  |  | |  |

**Supplementary** **Table 5. Single Nucleotide Polymorphism (SNP), Haplotype, and Diplotype Analysis in Breast and**

**Prostate Cancer Cohort Consortium (BPC3)**

| ***ERCC6* SNP** | **Alleles** | **Cases (N=977)** | **Cases %** | **Controls (N=1026)** | **Controls %** | **OR (95% CI)** | **P-Value** |  |
| --- | --- | --- | --- | --- | --- | --- | --- | --- |
| **rs3750751** | GG | 812 | 83.11 | 879 | 85.67 | 1 |  |  |
|  | **GA** | **161** | **16.48** | **143** | **13.94** | **1.348 (1.011 - 1.797)** | **0.042** |  |
|  | AA | 4 | 0.41 | 4 | 0.39 | 2.901 (0.524 - 16.071) | 0.223 |  |
| rs2228529 | AA | 560 | 57.32 | 594 | 57.89 | 1 |  |  |
|  | AG | 355 | 36.34 | 351 | 34.21 | 1.000 (0.804 - 1.243) | 0.998 |  |
|  | GG | 61 | 6.24 | 69 | 6.73 | 0.857 (0.565 - 1.301) | 0.469 |  |
| rs4838519 | AA | 243 | 24.87 | 271 | 26.41 | 1 |  |  |
|  | AC | 486 | 49.74 | 512 | 49.90 | 0.967 (0.757 - 1.236) | 0.788 |  |
|  | CC | 247 | 25.28 | 243 | 23.68 | 1.251 (0.936 - 1.673) | 0.130 |  |
| rs2228527 | AA | 560 | 57.32 | 594 | 57.89 | 1 |  |  |
|  | AG | 356 | 36.44 | 362 | 35.28 | 0.966 (0.778 - 1.200) | 0.756 |  |
|  | GG | 61 | 6.24 | 70 | 6.82 | 0.826 (0.546 - 1.249) | 0.365 |  |
| rs2229760 | CC | 364 | 37.26 | 384 | 37.43 | 1 |  |  |
|  | CT | 454 | 46.47 | 488 | 47.56 | 0.875 (0.700 - 1.094) | 0.242 |  |
|  | TT | 153 | 15.66 | 153 | 14.91 | 1.146 (0.840 - 1.562) | 0.390 |  |
| rs3750749 | TT | 855 | 87.51 | 880 | 85.77 | 1 |  |  |
|  | TC | 121 | 12.38 | 139 | 13.55 | 0.856 (0.636 - 1.153) | 0.307 |  |
|  | CC | 1 | 0.10 | 7 | 0.68 | 0.201 (0.023 - 1.731) | 0.144 |  |
| rs7076173 | TT | 775 | 79.32 | 807 | 78.65 | 1 |  |  |
|  | TC | 187 | 19.14 | 203 | 19.79 | 0.953 (0.734 - 1.239) | 0.721 |  |
|  | CC | 13 | 1.33 | 16 | 1.56 | 1.156 (0.495 - 2.701) | 0.733 |  |
| rs4253126 | CC | 777 | 79.53 | 833 | 81.19 | 1 |  |  |
|  | CA | 191 | 19.55 | 183 | 17.84 | 1.104 (0.849 - 1.436) | 0.461 |  |
|  | AA | 9 | 0.92 | 10 | 0.97 | 1.124 (0.388 - 3.254) | 0.824 |  |
| rs971667 | GG | 559 | 57.22 | 593 | 57.80 | 1 |  |  |
|  | GA | 357 | 36.54 | 363 | 35.38 | 0.972 (0.783 - 1.208) | 0.799 |  |
|  | AA | 61 | 6.24 | 70 | 6.82 | 0.828 (0.547 - 1.253) | 0.372 |  |
|  |  |  |  |  |  |  |  |  |
| rs4838524 | TT | 776 | 79.43 | 805 | 78.46 | 1 |  |  |
|  | TC | 188 | 19.24 | 205 | 19.98 | 0.943 (0.727 - 1.224) | 0.661 |  |
|  | CC | 13 | 1.33 | 16 | 1.56 | 1.152 (0.493 - 2.691) | 0.744 |  |
| rs958967 | AA | 777 | 79.53 | 804 | 78.36 | 1 |  |  |
|  | AG | 187 | 19.14 | 205 | 19.98 | 0.933 (0.719 - 1.212) | 0.605 |  |
|  | GG | 13 | 1.33 | 16 | 1.56 | 1.149 (0.492 - 2.685) | 0.748 |  |
| rs4253077 | GG | 818 | 83.73 | 842 | 82.07 | 1 |  |  |
|  | GT | 154 | 15.76 | 174 | 16.96 | 0.967 (0.732 - 1.278) | 0.815 |  |
|  | TT | 5 | 0.51 | 10 | 0.97 | 0.552 (0.164 - 1.853) | 0.336 |  |
| rs4253060 | GG | 552 | 56.50 | 601 | 58.58 | 1 |  |  |
|  | GA | 70 | 7.16 | 90 | 8.77 | 0.780 (0.513 - 1.185) | 0.244 |  |
|  | AA | 0 | 0.00 | 3 | 0.29 | N/A | N/A |  |
| rs2228528 | GG | 665 | 68.07 | 699 | 68.13 | 1 |  |  |
|  | GA | 291 | 29.79 | 297 | 28.95 | 0.988 (0.789 - 1.236) | 0.913 |  |
|  | AA | 21 | 2.15 | 30 | 2.92 | 0.821 (0.432 - 1.562) | 0.548 |  |
| rs4253042 | AA | 561 | 57.42 | 595 | 57.99 | 1 |  |  |
|  | AG | 355 | 36.34 | 360 | 35.09 | 0.965 (0.777 - 1.199) | 0.750 |  |
|  | GG | 61 | 6.24 | 71 | 6.92 | 0.826 (0.546 - 1.249) | 0.364 |  |
| rs3793786 | TT | 535 | 54.76 | 540 | 52.63 | 1 |  |  |
|  | TC | 383 | 39.20 | 419 | 40.84 | 0.940 (0.760 - 1.163) | 0.570 |  |
|  | CC | 57 | 5.83 | 67 | 6.53 | 0.874 (0.569 - 1.340) | 0.536 |  |
| rs4253082 | GG | 666 | 68.17 | 698 | 68.03 | 1 |  |  |
|  | GA | 290 | 29.68 | 297 | 28.95 | 1.241 (0.645 - 2.384) | 0.518 |  |
|  | AA | 21 | 2.15 | 31 | 3.02 | 1.268 (0.670 - 2.398) | 0.465 |  |
| Unconditional logistic regression models adjusted for family history of breast cancer and cohort (i.e., consent group) and restricted to Caucasian ≥50 years of age | | | | | | | | |

| ***ERCC8* SNP** | **Alleles** | **Cases (N=977)** | **Cases %** | **Controls (N=1026)** | **Controls %** | **OR (95% CI)** | **P-Value** |
| --- | --- | --- | --- | --- | --- | --- | --- |
| rs4647128 | AA | 878 | 89.87 | 935 | 91.13 | 1 |  |
|  | AG | 89 | 8.84 | 83 | 8.09 | 1.118 (0.781 - 1.599) | 0.544 |
|  | GG | 0 | 0.00 | 3 | 0.29 | NA | NA |
| rs158570 | AA | 343 | 35.11 | 369 | 35.96 | 1 |  |
|  | AG | 440 | 45.04 | 478 | 46.59 | 0.960 (0.763 - 1.207) | 0.724 |
|  | GG | 180 | 18.42 | 171 | 16.67 | 1.064 (0.788 - 1.438) | 0.686 |
| rs12520314 | TT | 887 | 90.79 | 940 | 91.62 | 1 |  |
|  | TC | 65 | 6.65 | 69 | 6.73 | 1.112 (0.737 - 1.677) | 0.614 |
|  | CC | 1 | 0.10 | 2 | 0.19 | 1.188 (0.072 - 19.504) | 0.904 |

Unconditional logistic regression models adjusted for family history of breast cancer and consent group [i.e., three cohorts that make up our BPC3 dataset, namely Prostate, Lung, and Colorectal Cancer (PLCO), European Prospective Investigation into Cancer and Nutrition (EPIC), and Polish Breast Cancer Study (PBCS) and restricted to Caucasian ≥50 years of age

| ***ERCC6* Haplotype** | **Haplotype  number** | **Cases  (N=1954)** | **Cases %** | | **Controls  (N=2052)** | | **Controls %** | | **OR (95% CI)** | | | **p-value** | | |  |  |
| --- | --- | --- | --- | --- | --- | --- | --- | --- | --- | --- | --- | --- | --- | --- | --- | --- |
| GACATTTCGTAGGGGAT | 1 | 758 | 38.79 | | 794 | | 38.69 | | 1 | | | 1 | | |  |  |
| GGAGCTTCATAGGGGGT | 2 | 476 | 24.36 | | 501 | | 24.42 | | 0.943 (0.784-1.135) | | | 0.534 | | |  |  |
| GAAACTTAGTAAGGAAC | 3 | 208 | 10.64 | | 202 | | 9.84 | | 1.074 (0.833-1.385) | | | 0.581 | | |  |  |
| GAAACTCCGCGGTGGAC | 4 | 164 | 8.39 | | 192 | | 9.36 | | 0.939 (0.717-1.231) | | | 0.650 | | |  |  |
| GAAACCTCGTAAGAAAC | 5 | 122 | 6.24 | | 153 | | 7.46 | | 0.811 (0.604-1.089) | | | 0.164 | | |  |  |
| AACACTTCGTAGGGGAT | 6 | 166 | 8.50 | | 151 | | 7.36 | | 1.300 (0.978-1.728) | | | 0.071 | | |  |  |
| GACACTCCGCGGGGGAT | 7 | 48 | 2.46 | | 43 | | 2.10 | | 1.130 (0.675-1.892) | | | 0.641 | | |  |  |
| GACACTTCGTAGGGGAT | 8 | 3 | 0.15 | | 8 | | 0.39 | | 0.651 (0.154-2.752) | | | 0.559 | | |  |  |
| Rare* | 9 | 9 | 0.46 | | 8 | | 0.39 | | 1.709 (0.598-4.880) | | | 0.317 | | |  |  |
| Unconditional logistic regression models adjusted for family history of breast cancer and consent group [i.e., three cohorts that make up our BPC3 dataset, namely Prostate, Lung, and Colorectal Cancer (PLCO), European Prospective Investigation into Cancer and Nutrition (EPIC), and Polish Breast Cancer Study (PBCS) and restricted to Caucasian ≥50 years of age  *Rare haplotypes with individual frequencies of <2% among the controls: | | | | | | | | | | | | | | |  |  |
| GAAACTTCGCGGTGGAC |  |  |  |  |  |  |  |  |  |  |  |  |  |  |  |  |
| GAAACTTCGTAAGGGAC |  |  |  |  |  |  |  |  |  |  |  |  |  |  |  |  |
| GAAACTTAATAAGGAAC |  |  |  |  |  |  |  |  |  |  |  |  |  |  |  |  |
| GACATTTCGTAAGAAAC |  |  |  |  |  |  |  |  |  |  |  |  |  |  |  |  |
| GGAGCTTCATAGGGGAT |  |  |  |  |  |  |  |  |  |  |  |  |  |  |  |  |
| GGCACTTCGTAGGGGGT |  |  |  |  |  |  |  |  |  |  |  |  |  |  |  |  |
| GAAACTTCGTAAGGGGC |  |  |  |  |  |  |  |  |  |  |  |  |  |  |  |  |
| GAAACCTCATAAGAAAC |  |  |  |  |  |  |  |  |  |  |  |  |  |  |  |  |
| GACATTCCGCGGGGGAT |  |  |  |  |  |  |  |  |  |  |  |  |  |  |  |  |
| GACATTTCGTAGGAAAC |  |  |  |  |  |  |  |  |  |  |  |  |  |  |  |  |
| GGAGCTTCACAGGGGGT |  |  |  |  |  |  |  |  |  |  |  |  |  |  |  |  |
| GGCGCTTCGTAGGGGAT |  |  |  |  |  |  |  |  |  |  |  |  |  |  |  |  |
| AACATTTCGTAGGGGAT |  |  |  |  |  |  |  |  |  |  |  |  |  |  |  |  |
| ***ERCC6* Diplotype** | | | | | **Diplotype number** | | **Cases (N=977)** | | **Cases %** | | **Controls (N=1026)** | **Controls %** | | **OR (95% CI)** | **p-value** | |
| GACATTTCGTAGGGGAT,GACATTTCGTAGGGGAT | | | | | 1,1 | | 151 | | 15.46 | | 153 | 14.91 | | 1 | 1 | |
| GACATTTCGTAGGGGAT,GGAGCTTCATAGGGGGT | | | | | 1,2 | | 177 | | 18.12 | | 177 | 17.25 | | 0.774 (0.543-1.104) | 0.157 | |
| GAAACTTAGTAAGGAAC,GACATTTCGTAGGGGAT | | | | | 3,1 | | 85 | | 8.70 | | 80 | 7.80 | | 0.880 (0.565-1.371) | 0.572 | |
| GAAACTCCGCGGTGGAC,GACATTTCGTAGGGGAT | | | | | 4,1 | | 58 | | 5.94 | | 75 | 7.31 | | 0.695 (0.423-1.145) | 0.153 | |
| GAAACCTCGTAAGAAAC,GACATTTCGTAGGGGAT | | | | | 5,1 | | 47 | | 4.81 | | 71 | 6.92 | | 0.513 (0.311-0.847) | 0.009 | |
| GGAGCTTCATAGGGGGT,GGAGCTTCATAGGGGGT | | | | | 2,2 | | 61 | | 6.24 | | 70 | 6.82 | | 0.723 (0.451-1.159) | 0.178 | |
| GACATTTCGTAGGGGAT,AACACTTCGTAGGGGAT | | | | | 1,6 | | 63 | | 6.45 | | 65 | 6.34 | | 0.991 (0.606-1.621) | 0.972 | |
| GAAACTCCGCGGTGGAC,GGAGCTTCATAGGGGGT | | | | | 4,2 | | 44 | | 4.50 | | 49 | 4.78 | | 0.927 (0.542-1.584) | 0.781 | |
| GAAACTTAGTAAGGAAC,GGAGCTTCATAGGGGGT | | | | | 3,2 | | 45 | | 4.61 | | 49 | 4.78 | | 0.918 (0.536-1.573) | 0.756 | |
| GAAACCTCGTAAGAAAC,GGAGCTTCATAGGGGGT | | | | | 5,2 | | 36 | | 3.68 | | 35 | 3.41 | | 0.902 (0.500-1.627) | 0.732 | |
| GGAGCTTCATAGGGGGT,AACACTTCGTAGGGGAT | | | | | 2,6 | | 43 | | 4.40 | | 34 | 3.31 | | 1.116 (0.619-2.011) | 0.715 | |
| GAAACTCCGCGGTGGAC,GAAACTTAGTAAGGAAC | | | | | 4,3 | | 22 | | 2.25 | | 20 | 1.95 | | 0.868 (0.406-1.854) | 0.714 | |
| GAAACTCCGCGGTGGAC,AACACTTCGTAGGGGAT | | | | | 4,6 | | 14 | | 1.43 | | 16 | 1.56 | | 0.742 (0.310-1.773) | 0.502 | |
| GACACTCCGCGGGGGAT,GACATTTCGTAGGGGAT | | | | | 7,1 | | 18 | | 1.84 | | 15 | 1.46 | | 0.851 (0.350-2.067) | 0.721 | |
| GAAACTTAGTAAGGAAC,GAAACCTCGTAAGAAAC | | | | | 3,5 | | 18 | | 1.84 | | 13 | 1.27 | | 0.819 (0.324-2.066) | 0.672 | |
| GAAACTTAGTAAGGAAC,AACACTTCGTAGGGGAT | | | | | 3,6 | | 11 | | 1.13 | | 13 | 1.27 | | 1.468 (0.607-3.549) | 0.394 | |
| GAAACCTCGTAAGAAAC,AACACTTCGTAGGGGAT | | | | | 5,6 | | 15 | | 1.54 | | 11 | 1.07 | | 1.399 (0.547-3.576) | 0.483 | |
| GACACTCCGCGGGGGAT,GGAGCTTCATAGGGGGT | | | | | 7,2 | | 6 | | 0.61 | | 11 | 1.07 | | 0.431 (0.142-1.311) | 0.138 | |
| GAAACTTAGTAAGGAAC,GAAACTTAGTAAGGAAC | | | | | 3,3 | | 5 | | 0.51 | | 10 | 0.97 | | 0.946 (0.318-2.815) | 0.921 | |
| GAAACTCCGCGGTGGAC,GAAACTCCGCGGTGGAC | | | | | 4,4 | | 9 | | 0.92 | | 10 | 0.97 | | 0.477 (0.139-1.642) | 0.241 | |
| Rare* | | | | |  | | 49 | | 5.02 | | 49 | 4.78 | | 1.231 (0.719-2.106) | 0.449 | |

Unconditional logistic regression models adjusted for family history of breast cancer and consent group [i.e., three cohorts that make up our BPC3 dataset, namely Prostate, Lung, and Colorectal Cancer (PLCO), European Prospective Investigation into Cancer and Nutrition (EPIC), and Polish Breast Cancer Study (PBCS) and restricted to Caucasian ≥50 years of age

*Rare diplotypes with individual frequencies of <2% among the controls:

| GACATTTCGTAGGGGAT,AACATTTCGTAGGGGAT |
| --- |
| GAAACTTCGTAAGGGAC,GAAACCTCGTAAGAAAC |
| GAAACTTCGTAAGGGAC,AACACTTCGTAGGGGAT |
| GACATTTCGTAAGAAAC,AACACTTCGTAGGGGAT |
| GAAACTTAATAAGGAAC,GACATTTCGTAGGGGAT |
| GACATTCCGCGGGGGAT,GACATTTCGTAGGGGAT |
| GACATTTCGTAGGAAAC,GGAGCTTCATAGGGGGT |
| GGAGCTTCACAGGGGGT,AACACTTCGTAGGGGAT |
| GGAGCTTCATAGGGGGT,GGCACTTCGTAGGGGGT |
| GGAGCTTCATAGGGGGT,AACATTTCGTAGGGGAT |
| GAAACCTCATAAGAAAC,GACATTTCGTAGGGGAT |
| GAAACTTAGTAAGGAAC,GACACTCCGCGGGGGAT |
| GAAACTCCGCGGTGGAC,GAAACCTCGTAAGAAAC |
| GAAACTCCGCGGTGGAC,GACACTCCGCGGGGGAT |
| GAAACTCCGCGGTGGAC,GACACTTCGTAGGGGAT |
| GAAACCTCGTAAGAAAC,GAAACCTCGTAAGAAAC |
| GAAACCTCGTAAGAAAC,GACACTCCGCGGGGGAT |
| AACACTTCGTAGGGGAT,AACACTTCGTAGGGGAT |
| GACACTCCGCGGGGGAT,AACACTTCGTAGGGGAT |
| GACACTCCGCGGGGGAT,GACACTCCGCGGGGGAT |
| GACACTTCGTAGGGGAT,GACATTTCGTAGGGGAT |
| GACACTTCGTAGGGGAT,GGAGCTTCATAGGGGGT |
| GACACTTCGTAGGGGAT,AACACTTCGTAGGGGAT |
| GAAACTTCGCGGTGGAC,GACATTTCGTAGGGGAT |
| GAAACTTCGCGGTGGAC,GGAGCTTCATAGGGGGT |

| ***ERCC8* Haplotype** | **Haplotype number** | **Cases (N=1954)** | **Cases %** | **Controls  (N=2052)** | **Controls %** | **OR (95% CI)** | **p-value** |  |
| --- | --- | --- | --- | --- | --- | --- | --- | --- |
| AAT | 1 | 1073 | 54.91 | 1151 | 56.09 | 1 | 1 |  |
| AGT | 2 | 725 | 37.10 | 739 | 36.01 | 1.026 (0.881-1.195) | 0.739 |  |
| GGT | 3 | 89 | 4.55 | 89 | 4.34 | 1.059 (0.746-1.503) | 0.748 |  |
| AAC | 4 | 67 | 3.43 | 73 | 3.56 | 1.110 (0.743-1.658) | 0.610 |  |
| Unconditional logistic regression models adjusted for family history of breast cancer and consent group [i.e., three cohorts that make up our BPC3 dataset, namely Prostate, Lung, and Colorectal Cancer (PLCO), European Prospective Investigation into Cancer and Nutrition (EPIC), and Polish Breast Cancer Study (PBCS) and restricted to Caucasian ≥50 years of age | | | | | | | | |

| ***ERCC8* Diplotype** | **Diplotype number** | **Cases (N=977)** | **Cases %** | **Controls (N=1026)** | **Controls %** | **OR (95% CI)** | **p-value** |  |
| --- | --- | --- | --- | --- | --- | --- | --- | --- |
| AAT,AAT | 1,1 | 304 | 31.12 | 323 | 31.48 | 1 | 1 |  |
| AGT,AAT | 2,1 | 380 | 38.89 | 413 | 40.25 | 0.982 (0.769-1.253) | 0.882 |  |
| AGT,AGT | 2,2 | 142 | 14.53 | 136 | 13.26 | 1.026 (0.735-1.431) | 0.881 |  |
| AAT,GGT | 1,3 | 47 | 4.81 | 48 | 4.68 | 0.951 (0.575-1.573) | 0.846 |  |
| AAT,AAC | 1,4 | 38 | 3.89 | 44 | 4.29 | 1.066 (0.612-1.858) | 0.821 |  |
| AGT,GGT | 2,3 | 38 | 3.89 | 32 | 3.12 | 1.371 (0.779-2.414) | 0.274 |  |
| AGT,AAC | 2,4 | 23 | 2.35 | 22 | 2.14 | 1.160 (0.585-2.302) | 0.671 |  |
| Rare* |  | 5 | 0.51 | 8 | 0.78 | 0.744 (0.206-2.687) | 0.652 |  |
| Unconditional logistic regression models adjusted for family history of breast cancer and consent group (i.e., cohorts that make up our BPC3 dataset) and restricted to Caucasian subjects ≥50 years of age  *Rare diplotypes with individual frequencies of <2% among the controls: | | | | | | | | |
| GGT,GGT |  |  |  |  |  |  |  |  |
| GGT,AAC |  |  |  |  |  |  |  |  |
| AAC,AAC |  |  |  |  |  |  |  |  |
